# A hidden two-locus disease association pattern in genome-wide association studies

**DOI:** 10.1186/1471-2105-12-156

**Published:** 2011-05-14

**Authors:** Can Yang, Xiang Wan, Qiang Yang, Hong Xue, Nelson LS Tang, Weichuan Yu

**Affiliations:** 1 Department of Electronic and Computer Engineering, Hong Kong University of Science and Technology, Hong Kong; 2 Department of Computer Science, Hong Kong University of Science and Technology, Hong Kong; 3 Department of Biochemistry, Hong Kong University of Science and Technology, Hong Kong; 4 Laboratory for Genetics of Disease Susceptibility, Li Ka Shing Institute of Health Sciences, The Chinese University of Hong Kong, Hong Kong

## Abstract

**Background:**

Recent association analyses in genome-wide association studies (GWAS) mainly focus on single-locus association tests (marginal tests) and two-locus interaction detections. These analysis methods have provided strong evidence of associations between genetics variances and complex diseases. However, there exists a type of association pattern, which often occurs within local regions in the genome and is unlikely to be detected by either marginal tests or interaction tests. This association pattern involves a group of correlated single-nucleotide polymorphisms (SNPs). The correlation among SNPs can lead to weak marginal effects and the interaction does not play a role in this association pattern. This phenomenon is due to the existence of unfaithfulness: the marginal effects of correlated SNPs do not express their significant joint effects faithfully due to the correlation cancelation.

**Results:**

In this paper, we develop a computational method to detect this association pattern masked by unfaithfulness. We have applied our method to analyze seven data sets from the Wellcome Trust Case Control Consortium (WTCCC). The analysis for each data set takes about one week to finish the examination of all pairs of SNPs. Based on the empirical result of these real data, we show that this type of association masked by unfaithfulness widely exists in GWAS.

**Conclusions:**

These newly identified associations enrich the discoveries of GWAS, which may provide new insights both in the analysis of tagSNPs and in the experiment design of GWAS. Since these associations may be easily missed by existing analysis tools, we can only connect some of them to publicly available findings from other association studies. As independent data set is limited at this moment, we also have difficulties to replicate these findings. More biological implications need further investigation.

**Availability:**

The software is freely available at http://bioinformatics.ust.hk/hidden_pattern_finder.zip.

## Background

The development of DNA microchip technology has allowed the analysis of single nucleotide polymorphism (SNPs) on a genome-wide scale to identify genetic variants associated with diseases. Researchers have proposed many methods to investigate association patterns of complex diseases. Two recent reviews [1,2] presented detailed analyses on many popular methods and tools, such as multifactor dimensionality reduction (MDR) [3], Random Jungle [4], Bayesian epistasis association mapping (BEAM) [5] and PLINK [6]. MDR is a popular non-parametric approach for detecting all possible *k*-way combinations of SNPs that interact to influence disease traits. Random Jungle (i.e., Random Forest [7]), is to solve classification and regression problems. In random forest, decision trees are combined to produce accurate predication. Its ability to handle the high dimensional problems in GWAS has been shown in [8,9]. BEAM designs a Bayesian marker partition model which classifies SNP markers into three types: SNPs unassociated with the disease, SNPs contributing to the disease susceptibility independently, and SNPs influencing the disease risk jointly with each other. In this model, a first order Markov chain is designed for the accommodation of correlation between adjacent SNPs. Markov Chain Monte Carlo (MCMC) sampling is used to optimize the posterior probability of the model. In addition, the "B-statistic" designed in BEAM can be used in the frequentist hypothesis-testing framework. PLINK provides a toolkit for flexible analyses, in which various statistical tests for single-locus analysis, haplotype analysis and allelic-based interaction analysis are implemented. Recently, a new method named "BOOST" [10] allows examination of all pairwise interactions in genome-wide case-control studies. As a result, many genetic susceptibility determinants have been mapped.

However, there is another type of association pattern, which often occurs within local regions in the genome and may not be detected by these methods. This association pattern involves multiple correlated SNPs with neither strong marginal effects nor strong interaction effects. But they can jointly display strong associations. Here we use some simple regression models to explain this association pattern. Suppose we have two dependent variables, *X*_1 _and *X*_2_, and one independent variable *Y*. We can fit two regression models (or logistic regressions for case-control data),  and , to test the association significance of these two variables. Here  and  are named as marginal coefficients. If these two marginal coefficients are very small, single variable analysis methods will consider them statistically insignificant and ignore them.

**Figure 1:**
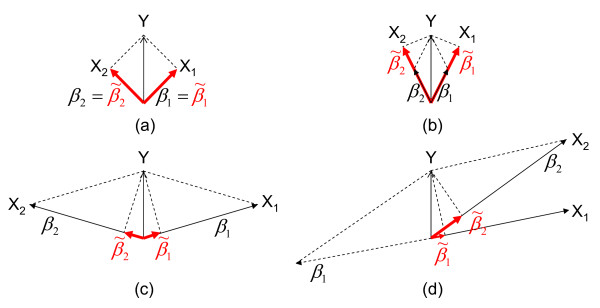
**Illustration of unfaithfulness in association studies**. There are three regression models in each scenario: *Y *~ *β*_1_*X*_1 _+ *β*_2_*X*_2_,  and . In this figure, the marginal coefficient  and  are shown as projections (marked with bold red color) of *Y *on *X*_1 _and *X*_2_, respectively. (a) *X*_1 _is not correlated with *X*_2_. (b) *X*_1 _is positively correlated with *X*_2_. (c) *X*_1 _is negatively correlated with *X*_2_. (d) *X*_1 _is positively correlated with *X*_2 _but the sign of *β*_1 _is the opposite of the sign of *β*_2_. Scenario (c) and Scenario (d) illustrate unfaithfulness.

However, if *X*_1 _correlates with *X*_2_, fitting the model *Y *~ *β*_1_*X*_1 _+ *β*_2_*X*_2 _may identify a new association pattern with *β*_1 _and *β*_2 _(named as bivariate regression coefficients) being significantly larger than  and . This phenomenon is referred to as unfaithfulness. It means that the marginal effects of correlated variables do not express their significant joint effects faithfully due to the correlation cancelation [11]. Figure 1 provides some synthetic examples to show the unfaithfulness involving two variables. There are four scenarios to illustrate the relationship between marginal coefficients (marked using red color) and bivariate regression coefficients. The first scenario (Figure 1:(a)) is a reference case that involves no correlations between *X*_1 _and *X*_2_. The marginal coefficients  and  are equal to the bivariate regression coefficients *β*_1 _and *β*_2_, respectively. In the second scenario (Figure 1:(b)), *X*_1 _is positively correlated with *X*_2_. The marginal coefficients are bigger than the bivariate regression coefficients. In the third scenario (Figure 1:(c)), *X*_1 _is negatively correlated with *X*_2_. The marginal coefficient  and  could be significantly smaller than the bivariate regression coefficients *β*_1 _and. *β*_2 _In the the fourth scenario (Figure 1:(d)), *X*_1 _is positively correlated with *X*_2_. But the sign of *β*_1 _is the opposite of the sign of β_2_. The correlation effect in the third scenario and the fourth scenario causes the unfaithfulness. In mathematics, the relationship between the marginal coefficients and the bivariate regression coefficients is formulated as(1)

where  is the expectation of the marginal coefficient , *ρ*(*X*_1_, *X*_2_) is the population correlation between *X*_1 _and *X*_2_. The marginal coefficients depend on their bivariate regression coefficients as well as the variable correlation, as we illustrated in Figure 1. We will give more explanation on this relationship in the high dimensional setting in the discussion section.

From the statistical point of view, different correlation patterns could cause the marginal coefficients  and  significantly different from the bivariate regression coeffcients *β*_1 _and *β*_2 _(shown in Figure 1). In fact, the issue of unfaithfulness has been discussed in the causality literature [12]. In GWAS, the correlation among SNPs arises due to the linkage disequilibrium pattern of the genome. A natural question arises: *Does the issue of unfaithfulness occur in GWAS?*

To answer this question, a computational method for detecting associations masked by unfaithfulness is necessary. In this paper, we propose a simple method to detect such associations involving two SNPs. It can evaluate each SNP pair in genome-wide case-control studies in a fast manner. We have applied our method to analyze seven data sets from the Wellcome Trust Case Control Consortium (WTCCC). The experimental results show that these associations widely exist in GWAS. In this work, we only handle the unfaithfulness issue involving two SNPs while the unfaithfulness can exist among a large number of markers. The detection of associations involving three or more SNPs is too time-consuming and beyond the scope of this work.

## Results

### Experiment on simulation study

The simulation study is designed to compare our proposed method with other three methods for detecting associations in the presence of unfaithfulness. These three methods include the marginal association test (single-locus analysis), Lasso [11,13] and BEAM [5]. The reasons that we choose these methods for comparison are as follows:

• Marginal association test is used in almost every GWAS due to its simplicity and effectiveness.

• Lasso is a shrinkage and selection method for (generalized) linear regression. It imposes a sparsity constraint (i.e., only a small fraction of variables are relevant) and uses *L*_1 _penalty to eliminate irrelevant variables. Fast algorithms are available for Lasso. Thus, it can simultaneously analyze a huge number of variables. It is very popular in genetics [11,14-16].

• BEAM has the capability of detecting both marginal associations and interactions in large-scale data sets. It uses first order Markov chain to accommodate the correlation between adjacent SNPs.

The details about the parameter settings in simulation are provided in the method section. In our simulation study, we only handle the unfaithfulness involving two associated variables *X*_1 _and *X*_2 _by using β_1 _> 0, *β*_2 _< 0 and *ρ*(*X*_1_, *X*_2_) > 0 as illustrated in Figure 1(d). The marginal coefficients  and  will be small due to the cancelation given by Equation (1). When β_1 _> 0, *β*_2 _> 0 and *ρ*(*X*_1_, *X*_2_) < 0 the unfaithfulness also happens. This corresponds to a situation that the minor alleles of both *X*_1 _and *X*_2 _increase the diseases risk but *X*_1 _and *X*_2 _are negatively correlated, as illustrated in Figure 1(c).

**Figure 2:**
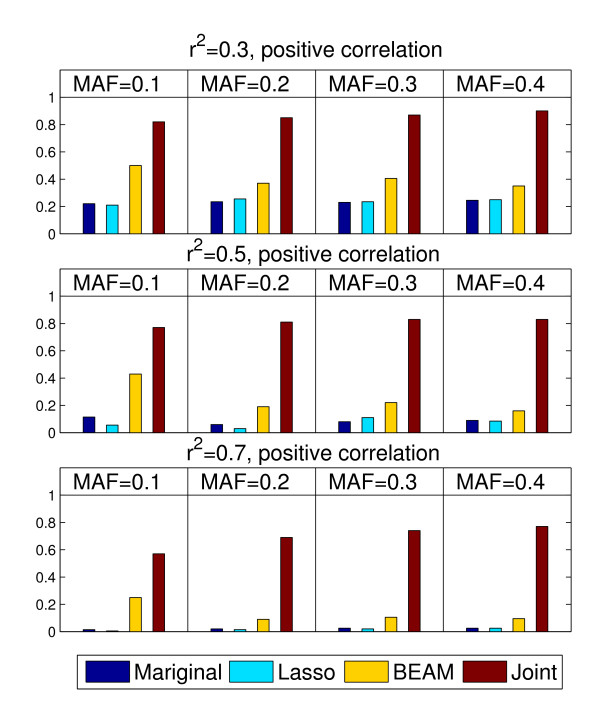
**The performance comparison of four methods: Marginal association tests, Lasso, BEAM and the proposed exhaustive two-locus joint analysis**. 100 data sets are generated under each parameter setting. 1000 samples (500 cases and 500 controls) are simulated in each data set. The power is calculated as the proportion of the 100 data sets in which the disease associated SNPs are detected.

The results in Figure 2 indicate that it is difficult for existing methods to detect the association masked by unfaithfulness while our proposed method achieves reasonable performance. Specifically, the poor performance of the marginal association test is not surprising since the marginal effects are weak in the presence of unfaithfulness. Although Lasso can simultaneously analyze all SNPs, it still suffers from the difficulty of detecting associations masked by unfaithfulness. This agrees with the analysis result in [11]. BEAM has a better performance, which should be attributed to its first order Markov chain designed for the accommodation of correlation. But its performance is still not comparable with the performance of our proposed method in most settings.

Another interesting point is that the statistical power of existing methods decreases as the linkage disequilibrium (LD) *r*^2 ^increases. Although our proposed method also degrades its performance when LD increases, it maintains a relatively high power for strong LD (*r*^2 ^= 0.7).

### Experiment on seven data sets from WTCCC

We have applied our method to analyze the data sets (14,000 cases in total and 3,000 shared controls) from the WTCCC [17]. WTCCC studies seven common human diseases, including bipolar disorder (BD), coronary artery disease (CAD), Crohn's disease (CD), hypertension (HT), rheumatoid arthritis (RA), type 1 diabetes (T1D) and type 2 diabetes (T2D). These data sets are generated using the affymetrix 500 K chip. We first apply a similar quality control procedure as suggested in [17] to pre-process the data. The numbers of remaining SNPs for seven data sets are around 360,000. In current stage, BEAM cannot directly handle these data sets [2].

Table 1 lists the numbers of identified two-locus associations masked by unfaithfulness under three statistical significance thresholds with and without the distance threshold for seven data sets. It shows that the unfaithfulness widely exists in these data sets. Some associations masked by unfaithfulness involve SNPs with at least 1 M base pair distance. However, all of them are located in the major histocompatibility complex (MHC) region (The MHC region encodes a large number of genes. It has extensive polymorphism and linkage disequilibrium with the long distance [18]). Therefore, the results in Table 1 provide the evidence that this association pattern typically occurs in local area. These results also suggest that using the local search can speed up the whole process in the future.

**Table 1:** The number of two-locus unfaithfulness associations identified from seven diseases data sets under different constraints.

	**BD**	**CAD**	**CD**	**HT**	**RA**	**T1D**	**T2D**
	
*T*^1^	48	31	25	46	132	153	67
	
*T*^2^	52	35	28	51	153	204	80
	
*T*^3^	60	36	29	52	165	252	84
	
*T*^1 ^& Dist	0	0	0	1	1	1	0
	
*T*^2 ^& Dist	0	0	0	3	17	17	0
	
*T *^3 ^& Dist	0	0	0	0	4	35	0

*T*^1 ^- the threshold of Bonferroni-corrected *P*-value is 0.10; *T*^2 ^- the threshold of Bonferroni-corrected *P*-value is 0.20; *T*^3 ^- the threshold of Bonferroni-corrected *P*-value is 0.30; Dist - the physical distance threshold between two SNPs is at least 1 Mb. This threshold is used to see how many unfaithfulness associations involving two remote loci

From the identified associations, we further conduct the gene mapping and identify some suspicious genes closely related with the disease traits. Table 2 and Table 3 report the unadjusted single-locus *P*-values, the unadjusted joint *P*-values, the marginal coefficients and joint bivariate coefficients for these associations. The other details are listed in the supplementary document (Additional file 1). These identified associations coincide with Figure 1(d). To date, we can only connect some identified associations to publicly available results from other association studies. Many identified association patterns still remain unexplained. In the following, we explain the details of some associations that are confirmed by other studies.

**Table 2:** Some associations masked by unfaithfulness from the WTCCC data set.

Disease	SNP *X_p_*	Single-locus *P *-value	SNP *X_q_*	Single-locus *P *-value	Chr	Gene	Unfaithfulness *P *-value
BD	rs668860	0.053	rs10873672	0.245	1	MCOLN2	4.885 × 10^-15^
	rs668860	0.053	rs6691970	0.216	1	MCOLN2	6.217 × 10^-15^

CAD	rs7162070	0.867	rs16969478	0.160	15	FSIP1	5.551 × 10^-15^
	rs1876853	0.903	rs16969478	0.160	15	FSIP1	2.310 × 10^-13^
	rs8029602	0.853	rs16969478	0.160	15	FSIP1	5.274 × 10^-14^
	rs16969475	0.823	rs16969478	0.160	15	FSIP1	1.259 × 10^-13^

T1D	rs1058318	0.074	rs2252745	0.840	6	GNL1, PPP1R10	1.326 × 10^-12^

HT	rs2300390	0.460	rs12482676	0.061	21	RCAN1	2.442 × 10^-15^

**Table 3:** Regression coefficients of those associations listed in Table 2.

Disease	SNP *X_p_*	**(*z *Value)**	SNP *X_q_*	**(*z *Value)**	*r*^2^	*β*_1_(*z *Value)	*β*_2_(*z *Value)
BD	rs668860	0.0162 (0.392)	rs10873672	0.0679 (1.662)	0.961	-1.379 (-5.823)	1.402 (5.989)
	rs668860	0.0162 (0.392)	rs6691970	0.0706 (1.728)	0.958	-1.397 (-5.934)	1.421 (6.107)

CAD	rs7162070	0.0301 (0.482)	rs16969478	-0.108 (-1.732)	0.913	2.621 (5.671)	-2.637 (-5.720)
	rs1876853	0.0208 (0.331)	rs16969478	-0.108 (-1.732)	0.913	2.354 (5.538)	-2.372 (-5.603)
	rs8029602	0.0175 (0.281)	rs16969478	-0.108 (-1.732)	0.914	2.214 (5.594)	-2.238 (-5.676)
	rs16969475	0.0184 (0.294)	rs16969478	-0.108 (-1.732)	0.913	2.110 (5.660)	-2.132 (-5.746)

T1D	rs1058318	0.0992 (2.269)	rs2252745	-0.0167 (-0.375)	0.887	1.0292 (7.530)	-1.005 (-7.219)

HT	rs2300390	-0.0600 (-1.182)	rs12482676	0.0531 (1.037)	0.903	-1.215 (-6.567)	1.224 (6.577)

Here we assume additive effects in regression.

#### Bipolar disorder (BD)

Among associated SNP pairs identified from the BD data set, we find two suspicious SNP pairs, (rs668860, rs10873672) and (rs668860, rs6691970). The unadjusted *P*-values for these two SNP pairs are 4.885 × 10^-15 ^and 6.217 × 10^-15^, respectively. They are still significant after Bonferroni correction. However, none of these three SNPs (rs668860 is involved in both pairs) was reported in [17] because their marginal effects are too weak to be detected by the single-locus association test. The unadjusted *P*-values for these three SNPs based on the single-locus association test are 0.053, 0.245 and 0.216, respectively. All three SNPs reside in the intron of gene MCOLN2. The protein Mucolipin-2, encoded by gene MCOLN2 and also known as TRPML2 (transient receptor potential cation channel, mucolipin subfamily, member 2), has been confirmed to have strong associations with bipolar disorder in a family-based association study [19]. To our knowledge, this is the first identified association between the MCOLN2 gene and the bipolar disorder risk in a population-based association study.

**Figure 3:**
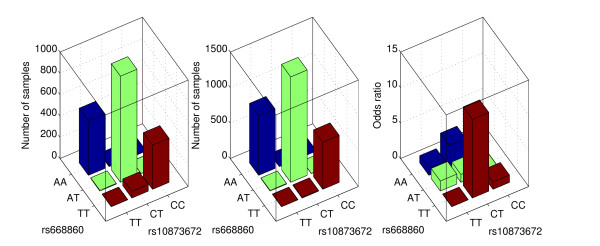
**Distributions of genotypes of rs668860 and rs10873672 in the bipolar disorder data set and the odds ratio computed for combined genotypes of these two SNPs**. Left Panel: The distribution of genotypes of rs668860 and rs10873672 in case samples. Middle Panel: The distribution of genotypes of rs668860 and rs10873672 in control samples. Right panel: The estimated odds ratio for the combination of rs668860 and rs10873672. The odds ratio of genotype combination "AA/TT" is used as reference. The genotype combination "TT/CT" has significantly higher odds ratio than other genotype combinations.

Figure 3 shows the joint distributions of the pair (rs668860, rs10873672) (The other pair shares a similar pattern.) in cases and controls and the corresponding odds ratios. The genotype combination "CT/TT" has a significantly higher odds ratio than other genotype combinations. Further investigations of the MCOLN2 gene may help identify the causes of bipolar disorder disease.

**Figure 4:**
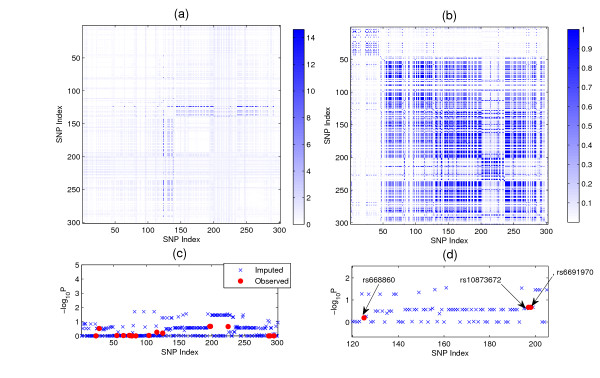
**Analysis result of the local region of the BD data set located by rs668860, rs10873672 and rs6691970**. (a) The enriched signal after imputation: The intensity shows -*log*_10_*P *given by the joint regression. (b) The LD structure of this local area. (c) The -*log*_10_*P *value obtained using single-SNP analysis. (d) The locations of rs668860, rs10873672 and rs6691970.

We further use BEAGLE [20] to impute the SNPs in this local area so that we can see the enriched signals after imputation. This region includes 300 SNPs. It begins with the SNP rs1030933 and ends with the SNP rs1837329. After imputation, we analyze the imputed data and the result is given in Figure 4. Figure 4(a) shows the enriched signal. The intensity shows -*log*_10_*P *values given by the joint regression (*P*-value is calculated based on ). Figure 4(b) shows the LD structure of this local area. Figure 4(c) shows the -*log*_10_*P *values obtained using single-SNP analysis (P-values are calculated based ). Figure 4(d) shows the locations of rs668860, rs10873672 and rs6691970. From Figure 4, we can see the following:

• Although this region is in strong LD (see Figure 4(b)), association masked by unfaithfulness does not happen across the entire area. This shows that this type of asssociation not only depends on the correlation structure but also depends on the effects of the SNPs, as we illustrated in Figure 1 (also see Equation 1).

• From Figure 4(c), the marginal effects of the imputed SNPs are very weak. This indicate that this type of association is not caused by some ungenotyped causative SNPs. Instead, it is a genuine effect.

#### Coronary artery disease (CAD)

We identify four suspicious associations involving five SNPs. The unadjusted *P*-values for these four associations range from 2.310 × 10^-13 ^to 5.551 × 10^-15^. The unadjusted single-locus *P*-values for five SNPs involved in these five associations indicate that they do not have noticeable marginal effects. All five SNPs reside in the intron of gene FSIP1 (fibrous sheath interacting protein 1). We have not found evidence to directly connect gene FSIP1 with the coronary artery disease. However, the LD analysis identifies a well studied gene THBS1 (thrombospondin-1), which is centromeric to gene FSIP1 and has been confirmed to increase the risk of coronary artery disease in many studies [21-23]. It would be of great interest to investigate gene FSIP1 in determining genetic susceptibility to coronary artery disease.

**Figure 5:**
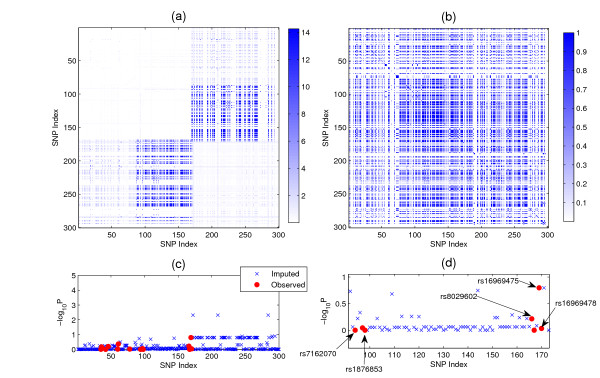
**Analysis result of the local region of the CAD data set located by rs7162070, rs1876853, rs8029602, rs16969475 and rs16969478**. (a) The enriched signal after imputation: The -*log*_10_*P *value given by the joint regression. (b) The LD structure (*r*^2^) in the same region. (c) The -*log*_10_*P *of single SNP analysis. (d) The locations of the genotyped SNPs rs7162070, rs1876853, rs8029602, rs16969475 and rs16969478.

Here we also show the enriched signals obtained from the imputation. Figure 5(a) shows the -*log*_10_*P *given by the joint regression. Figure 5(b) shows the LD structure (*r*^2^) in that region. Figure 5(c) shows the -*log*_10_*P *of single SNP analysis. Figure 5(d) shows the locations of the genotyped SNPs which are listed in Table 2. Again, the marginal effects of the imputed SNPs are weak. We see clearly that the signal of unfaithfulness appears in the block-like manner.

#### Type 1 diabetes (T1D)

Most identified associations from the T1D data set are linked with the MHC region. The MHC region at chromosomal position 6p21 encodes many genes (such as HLA-DQB1 and HLA-DRB1) that have been associated with type 1 diabetes [17,24] by using the single-locus test. However, it is still unclear which and how many loci within the MHC region determine T1D susceptibility because of the functional complexity of this small human genome segment. The MHC region has been connected with more than 100 diseases, such as diabetes, rheumatoid arthritis, psoriasis, asthma and various autoimmune disorders. Our results provide additional information to locate disease-associated loci. Concretely, one suspicious association involves SNP rs1058318 and SNP rs2252745. The unadjusted *P*-value of this association is 1.326 × 10^-12^. The unadjusted single-locus *P*-values of rs1058318 and rs2252745 are 0.074 and 0.840, respectively. SNP rs1058318 resides in the intron region of gene GNL1 and SNP rs2252745 resides in the intron region of gene PPP1R10. Both genes are located in the MHC region and adjacent to each other. Gene GNL1 belongs to the HLA-E class. The locus in HLA-E has been strongly associated with type 1 diabetes [25]. The detailed examination of the relationship between gene GNL1 and gene PPP1R10 may provide some new insights in studying the causes of type 1 diabetes.

#### Hypertension (HT)

Among associations identified from the HT data set, we find one suspicious pair involving SNP rs2300390 and SNP rs12482676. The unadjusted *P*-value is 2.442 × 10^-15^. The unadjusted single-locus *P*-values for rs2300390 and rs12482676 are 0.460 and 0.061, respectively. Both SNPs reside in the intron of gene RCAN1. Gene RCAN1 mainly functions as a regulator of calcineurin. Calcineurin participates in many cellular and tissue functions. Its abnormal expression is associated with many diseases including hypertension [26].

#### Crohn's disease (CD), rheumatoid arthritis (RA) and type 2 diabetes (T2D)

Currently, we have difficulties to connect the identified associations of CD, RA and T2D to publicly available findings from other association studies. Their biological implications need to be further explored.

### Experiment on the Illumina data sets from other independent studies

We further analyze the Crohn's Disease data set [27], in which 308,332 autosomal SNPs were assayed on the Illumina HumanHap300 chip. After a standard quality control (the proportion of miss values ≤ 10%, the minor allele frequency ≥ 5% and the *P*-value of Hardy-Weinberg equilibrium ≥ 0.0001), the number of remaining SNPs is 291,964.

We apply our method to this data set and do not find any significant associations masked by unfaithfulness. Our explanation is that Illumina chip uses the tagSNP design and the correlation among SNPs is less than that of Affymetrix 500 K chip used by WTCCC. This result indicates that it is unlikely to detect associations masked by unfaithfulness using the tagSNP design.

In order to check if imputation helps in identifying significant association masked by unfaithfulness, we focus on the SNP regions in which we have identified associations from the WTCCC CD data set (Additional file 1: Table S3), impute the corresponding SNP data from [27], and re-run our analysis. Unfortunately, we fail to replicate those findings in the WTCCC CD data set. We have examined the imputation result carefully. At those local regions we are interested in, few SNPs are directly genotyped. In the hapmap data, hundreds of SNPs locate in those areas. This implies hundreds of SNPs need to been imputed using the information coming from the reference panel. In fact, the frequencies of those imputed haplotypes are almost the same in cases and controls. This is probably the reason that we cannot replicate those findings. Hopefully, next-generation sequencing will provide high resolution SNP data to resolve this issue. Another important reason may be that these two CD data sets are from different populations (one comes from Europe, another comes from north America).

Similarly, we have analyzed another RA data set [28] from Genetic Analysis Workshop 16. This data set is acquired from North American population. The SNPs are genotyped by the Illumina chip. We also have difficulties to replicate the findings of the RA data set from WTCCC. We hope we can get access to more data sets to verify our results in the future.

## Discussion

### The unfaithfulness issue in the high dimensional feature space

In the high dimensional feature space, many features could correlate with each other by chance, which leads to the existence of unfaithfulness and poses a great challenge on feature selection and association analysis. In this work, we only handle the unfaithfulness issue involving two variables (SNPs), while the unfaithfulness can exist among a huge number of variables. The relationship between the marginal coefficient ( in ) and the regression coefficient ( in *β*_1_*X*_1_+···+*β_p_X_p_*+···+*β_s_X_s_*) is given as follows [11]:(2)

where  is the expectation of marginal coefficient, *ρ*(*X_q_*, *X_p_*) is the population correlation between *X*_*q *_and *X_p_*. If , then  can be close to zero no matter how large *β_p _*is. In addition, the number of involved variables could be very big. To exclude the effect of unfaithfulness in feature selection, Fan and Lv [29] had to make an assumption that there is a *C *> 0 such that  for *p *= 1,..., *s*, and then proved that the truly associated variables will be among those having the highest marginal coefficients.

In our simulation study, we only handle the unfaithfulness involving two associated variables *X*_1 _and *X*_2 _by using *β*_1 _> 0, *β*_2 _< 0 and *ρ*(*X*_1_, *X*_2_) > 0 as illustrated in Figure 1(d). The marginal coefficients  and  will be small due to the cancelation given by Equation (2). When *β*_1 _> 0, *β*_2 _> 0 and *ρ*(*X*_1_, *X*_2_) < 0, the unfaithfulness also happens. This corresponds to a situation that the minor alleles of both *X*_1 _and *X*_2 _increase the diseases risk but *X*_1 _and *X*_2 _are negatively correlated, as illustrated in Figure 1(c). The simulation result shows that the marginal test and Lasso perform poorly. The better performance of BEAM should be attributed to its first order Markov chain designed for the accommodation of correlation. Although our exhaustive method performs reasonably well, the direct extension of our method to deal with three or more loci is computationally expensive. Therefore, solving the unfaithfulness issue is still challenging.

### The Relationship between interaction models and unfaithfulness

In this work, we only deal with a two-locus association pattern involving the unfaithfulness. There are many works [3,5,6,30] discussing two-locus associations. Most of them belong to the category of interaction analysis (see details in [1,2]). The SNP interaction is also referred to as "epistasis". The most common statistical definition of interactions is the statistical deviation from the additive effects of two loci on the phenotype [2]. Using the same example we discussed in the introduction section, testing interactions between *X*_1 _and *X*_2 _is to first fit the regression model (or logistic regressions for case-control data) *Y *~ *β*_1_*X*_1 _+ *β*_2_*X*_2 _+ *β*_12_*X*_1_*X*_2 _and then test the significance of *β*_12_. There is no direct connection between *β*_1 _(or *β*_2_) and *β*_12 _In the analysis of unfaithfulness, the relationship between marginal coefficients (, ) and joint coefficients (*β*_1_, *β*_2_) is given in Equation (2). The interaction term plays no role here. Therefore, it is not appropriate to use interaction models to detect associations masked by unfaithfulness.

### The Relationship between unfaithfulness and confounding

Suppose we are studying the relationship between two variables *X *and *Y *using model . Confounding arises when there is another observed variable *Z *which is independently associated with *X *and *Y*. Specifically, we have  for model  and  fot model . When studying the relationship between *X *and *Y*, it is necessary to account for the confounding effect by using model *Y *~ *β_yz_Z *+ *β_yx_X*. In other words, confounding is more like the situation illustrated in Figure 1 (b). Readers are referred to [31] for the detailed explanation of confounding.

The unfaithfulness is different. For model  and , both  and  are close to zero. For joint model *Y *~ *β*_1_*X*_1 _+ *β*_2_*X*_2_, both *β*_1 _and *β*_2 _are not zero, as illustrated in Figure 1(c) and 1(d).

### Biological interpretations

There are two possible biological interpretations. The first interpretation is illustrated in Figure 1(d). Consider two loci *X_p _*and *X_q _*which are positively correlated. When *X_q _*increases the disease risk (*β_q _*> 0) and *X_p _*acts as a protective locus (*β_p _*< 0), unfaithfulness happens. The identified associations and their coefficients listed in Table 3 indicate that these associations indeed exist.

The second interpretation is illustrated in Figure 1(c). Consider two loci *X_p _*and *X_q _*which are negatively correlated. When both *X_p _*and *X_q _*increase the disease risk (*β_p _*> 0 and *β_q _*> 0), unfaithfulness also happens. This case may be particularly interesting when analyzing SNPs with low allele frequencies [32]. Suppose the allele frequencies of both *X_p _*and *X_q _*are low and thus the mutations happening at these two loci are relatively recent. We can further assume the haplotype *a *- *a *does not exist (because the probability of both two mutations happen in a short period is very small). This implies these two loci are negatively correlated. Unfortunately, we do not identify this type of associations. Possible reasons include: (1) The current genotyping chip is designed based on the "common disease/common variant" model [33,34], the low frequency SNPs are not directly assayed. (2) The statistical power of current testing strategy is relatively low to handle rare variants.

### The unfaithfulness implications on tagSNPs

GWAS is considered as a powerful approach to detecting genetic susceptibility of common diseases. Such studies require the genotypes of a huge number of SNPs across the genome, each of which is tested for association with the phenotype of interest. This is generally referred to as the direct test of association, in which the functional mutation is presumed to be genotyped. An alternative approach is to take advantage of the correlation between SNPs. This approach genotypes a subset of SNPs, called tagSNPs, which are in high linkage disequilibrium with other SNPs [33]. The association tests of tagSNPs are used to indirectly infer the association of other correlated SNPs. This approach is widely used to save genotyping costs in GWAS. Many tagging methods [33,35,36] have been developed to reduce the number of markers to be genotyped. One key assumption in these methods is that the association analysis of a set of highly correlated SNPs is equivalent with the association analysis of tagSNPs of this set. However, the existence of unfaithfulness poses a challenge for these methods. The weak marginal association of a tagSNP does not imply the weak association of the corresponding genome region in which this tagSNP is located. The reason is that some non-genotyped SNPs correlating with the tagSNPs may jointly display strong associations in the presence of unfaithfulness.

In this work, we analyzed the WTCCC data generated by the Affymetrix 500 K chip and a Crohn's disease data set generated by the Illumina chip. The Affymetrix 500 K chip spaces SNPs along the genome and the Illumina chip uses the tagSNP design. LD becomes less apparent in the Illumina data set and we did not find any association masked by unfaithfulness. This result suggests that it is very difficult to detect these associations by using the tagSNP design. If more SNPs could be genotyped in the future GWAS, we would detect more unknown associations.

## Conclusion

The phenomenon named "unfaithfulness" has been discussed as a mathematical concept in the literature. In this work, we answered the question whether there exist associations masked by unfaithfulness in genome-wide association studies. We developed a simple and fast method to examine all SNP pairs and demonstrated that our method is applicable to analyze genome-wide SNP data sets. We conducted experiments on both simulated data and seven real data sets from WTCCC and identify many associations masked by unfaithfulness. As expected, these identified associations only occur in local area. This implies that only the local search is needed to find such associations.

To date, we can only connect some identified associations to publicly available results from other association studies. As independent data set is limited as this moment, we have difficulties to replicate these findings. The biological interpretation of many findings remains unclear. It would be of great interest if their biological functions could be investigated. In addition, we only handle the two-locus associations in the presence of unfaithfulness. Detecting such associations for three or more loci is still an open problem.

## Methods

Given a data set with ℒ SNPs and *n *samples, we use *X_l _*to denote the *l*-th SNP, *l *= 1,···, ℒ and *Y *to denote the class label (0 for control and 1 for case). SNPs are bi-allelic genetic markers. Capital letters (e.g. *A, B*,...) and lowercase letters (e.g. *a, b*,...) are often used to denote major and minor alleles, respectively. For simplicity, we use {0, 1, 2} to represent the three genotypes {*AA*, *Aa*, *aa*}, respectively.

## Definition of the association masked by unfaithfulness

Considering a pair of loci *X_p _*and *X_q_*, four logistic regression models are typically involved to test associations masked by unfaithfulness:(3)(4)(5)

and(6)

where *I*(*V *= *v*) is the indicator function that takes the value 1 if *V *= *v *is true and 0 otherwise. In order to make the representation of both logistic regression models and log-linear models (introduced later) in a compact and consistent way, we use the notation adopted in [37] and rewrite the above logistic regression models in the following forms:(7)(8)(9)

and(10)

Please note that the superscripts *X_p _*and *X_q _*in Equation (8), Equation (9) and Equation (10) are merely the labels and do not represent the exponents. The term  represents the coefficient of *X_p _*at category *i*. Throughout this paper, we use ℳ to denote logistic regression models. We will use *M *to denote log-linear models. The log-likelihood function of a logistic model ℳ is denoted as *L*_ℳ_, and its maximum likelihood estimation (MLE) is denoted as . The log-likelihood function of a log-linear model *M *is denoted as *L_M_*, and its maximum likelihood estimation (MLE) is denoted as . For example, ℳ_1 _is a logistic regression model whose log-likelihood function and MLE are denoted by *L*_ℳ1 _and .

Our goal is to test if ℳ_1⊕2 _is significantly different from ℳ_0 _when both ℳ_1 _and ℳ_2 _are not. The likelihood ratio test is often used to conduct such tests. To test the difference between ℳ_1⊕2 _and ℳ_0_, the following three steps are involved:

1. Fit a logistic regression model defined in Equation (10) and obtain the log-likelihood .

2. Compute the log-likelihood  of the null logistic regression model defined in Equation (7).

3. Calculate *P*-value using the *χ*^2 ^test on the value 2 () with degree of freedom *df *= 2.

Similarly, the test of difference between ℳ_1 _(or ℳ_2_) and ℳ_0 _involves the following three steps:

1. Fit a logistic regression model defined in Equation (8) (or Equation (9)) to measure the main effect of *X_p _*(or *X_q_*) and obtain the log-likelihood  (or ).

2. Compute the log-likelihood  of the null logistic regression model defined in Equation (7).

3. Calculate *P*-value using the *χ*^2 ^test on the value 2 () (or 2 ()) with degree of freedom *df *= 2.

Directly using regression methods for testing all pairs of SNPs in GWAS would be very time-consuming. Often the parallel computation was recommended [38]. Here, we propose to use log-linear models [37] instead of logistical regression models in GWAS. We show that this makes the likelihood ratio test computationally more efficient in genome-wide SNP data analysis. In the following, we briefly summarize the key components. The details are explained in the supplementary document (Additional file 1).

### Likelihood ratio tests using log-linear models

Given two loci *X_p _*and *X_q_*, a contingency table of *X_p_*, *X*_*q*_, *Y *will be used to test the association masked by unfaithfulness between (*X*_*p*_, *X*_*q*_) and *Y*. The size of the contingency table is *I *× *J *× *K*, where *I *= 3, *J *= 3, *K *= 2. We use *n_ijk _*to denote the observed count in the cell (*i, j, k*) in the contingency table (Table 4). Here *n_ijk _*is considered as the realization of a random variable *N_ijk _*assumed as Poisson-distributed in log-linear models.

**Table 4:** The genotype counts in cases (*Y *= 1) and controls (*Y *= 2).

***Y *= 1**	***Xq *= 1**	***Xq *= 2**	***Xq *= 3**	***Y *= 2**	***Xq *= 1**	***Xq = *2**	***Xq = *3**
			
*Xp *= 1	*n*_111_	*n*_121_	*n*_131_	*Xp *= 1	*n*_112_	*n*_122_	*n*_132_
*Xp *= 2	*n*_211_	*n*_221_	*n*_231_	*Xp *= 2	*n*_212_	*n*_222_	*n*_232_
*Xp *= 3	*n*_311_	*n*_321_	*n*_331_	*Xp *= 3	*n*_312_	*n*_322_	*n*_332_

We use the dot convention to indicate summation over a subscript, e.g.,  is the number of observations with *X_p _= i*. Similarly, we have  and . We also have  and . Throughout this paper, we use  to denote the expectation of *N_ijk _*under log-linear model *M*, and use  to denote the MLE of .

The equivalence between log-linear models and logistic models are given in Table 5 (see model definitions in the supplementary document (Additional file 1)). Here we construct our test statistics based on three log-linear models, which are the homogeneous association model corresponding to the logistic regression model ℳ_1⊕2_, the partial independence model corresponding to the logistic regression model ℳ_1 _(or ℳ_2_), and the block independence model corresponding to the null logistic regression model ℳ_0_. Let , and  be the log-likelihood of the homogeneous association model *M_H_*, the partial independence model *M_P _*and the block independent model *M_B _*evaluated at their MLEs , and ,, respectively.

**Table 5:** Equivalence between log-linear models and logistic models for a three-way table with binary response variable *Y *(*M*_*B*_: Block independence model.

Log-linear model	Logistic model
	ℳ_0 _: *β*_0_
	
	

*M*_*P*_: Partial independence model. *M*_*H*_: Homogeneous association model).

Based on the equivalence, the deviance  of logistic regression models can be computed as(11)

and the deviance  (or ) can be computed as(12)

In Equation (11) and Equation (12),  and  have the closed-form solutions (please check the supplementary document (Additional file 1) for the derivation):(13)

and(14)

Iterative Proportional Fitting (IPF) [37] can be used to quickly estimate . Specifically, initialize  to be 1 for all *i, j, k*, then do IPF as follows:(15)

The updating formulas may only be ill-defined if , , or , due to multi-collinearity. If this happens, we set , (*m *= 1, 2,...) to zero accordingly. Our experimental results show that this solution works well in practice (We have compared our results with the standard software R, in which the multi-collinearity problem is elegantly handled when fitting generalized linear models. It turns out that our results agree with the results given by R). Then the test statistics can be efficiently computed. As a result, we are able to test every pair of loci to search for associations masked by unfaithfulness in GWAS. Table 6 gives the running time of our method for data sets of different sizes.

**Table 6:** Running time of the proposed method for data sets of different sizes.

Data size	Running time
*n *= 5, 000, ℒ = 1, 000	3s
*n *= 5, 000, ℒ = 5, 000	76s
*n *= 5, 000, ℒ = 10, 000	303s

All timings are carried out on one desktop computer with 3.0 GHz CPU and 4G memory running Windows XP professional x64 Edition system. Here *n *is the number of samples and ℒ is the number of SNPs.

### An exhaustive approach to detecting the two-locus associations masked by unfaithfulness in GWAS

This approach involves the following steps:

• Step 1. For all of ℒ SNP markers, we first filter out those SNPs with significant main effects using Equation (12) since we are only interested in those markers without significant main effects. The ℒ *P*-values can be adjusted by either the classic Benjamini-Hochberg method for controlling false discovery rate (FDR) or the Bonferroni correction for controlling family wise error rate (FWER).

• Step 2. For the remaining ℒ' SNPs without significant main effects, we check every pair using the Equation (11). Again, the *P*-values can be adjusted for controlling either FDR or FWER.

The *P*-value thresholds in both Step 1 and Step 2 are specified by users. The default setting of the threshold is 0.1. The multiple factor for Bonferroni correction is ℒ'(ℒ' - 1)/2, where ℒ' is the number of SNPs after removing those SNPs with significant marginal effects. Since the number of SNPs with significant marginal effects only accounts for a small fraction of the entire SNP set, we have ℒ'(ℒ' - 1)/2 ≈ ℒ(ℒ - 1)/2.

### Simulation design

Let *p*(*D*|*G_i_*) denote the probability of an individual being affected given its genotype *G_i _*(i.e., the penetrance of *G_i_*), and let  denote the probability of an individual not being affected given its genotype *G_i_*. Based on the definition of the odds of a disease(16)

the penetrance *p*(*D*|*G_i_*) of the genotype *G_i _*can be calculated using(17)

The disease prevalence *p*(*D*) and genetic heritability *h*^2 ^are given as(18)(19)

The odds table of our simulation model is given in Table 7. It is a multiplicative model of odds ratio, i.e., it is an additive model on the log-odds scale. The reason we choose this model is that we try to exclude interference of the interaction effect when we discuss the unfaithfulness. Essentially, the unfaithfulness arises due to the correlation cancelation. The interaction effects play no role here.

**Table 7:** The odds table of the simulation model.

**Model**	***BB***	***Bb***	***bb***	**Model**	***BB***	***Bb***	***bb***
			
*AA*	*α*	*αθ*_21_	*αθ*_21_*θ*_22_	*AA*	*α*	*αθ_b_*	
*Aa*	*αθ*_11_	*αθ*_11_*θ*_21_	*αθ*_11_*θ*_21_*θ*_22_	*Aa*	*αθ_a_*	*αθ_a _θ_b_*	
*aa*	*αθ*_11_*θ*_12_	*αθ*_11_*θ*_12_*θ*_21_	*αθ*_11_*θ*_12_*θ*_21_*θ*_22_	*aa*			

The parameters *α*, *θ*_11_, *θ*_12_, *θ*_21_, and *θ*_22 _control the prevalence *p*(*D*) (Equation(18)) and the heritability *h*^2 ^(Equation(19)). For simplicity, let *θ*_11 _= *θ*_12 _= *θ*_*a*_, *θ*_21 _= *θ*_22 _= *θ*_*b*_

For simplicity, we restrict *θ*_11 _= *θ*_12 _= *θ_a _*and *θ*_21 _= *θ*_22 _= *θ_b_*. The parameter *θ_a _*> 1 means that the minor allele "*a*" increases the disease risk. This corresponds to the bivariate regression coefficient *β*_1 _> 0. Similarly, *θ_b _*< 1 implies *β*_2 _< 0. In the presence of linkage disequilibrium (linkage disequilibrium measure Δ > 0), unfaithfulness arises. To simulate this situation, we further set *θ_a _*= *θ *and *θ_b _*= 1/*θ*. In the simulation, the prevalence *p*(*D*) and the heritability *h*^2 ^are controlled by the parameters *α *and *θ*. We first specify the disease prevalence *p*(*D*) and genetic heritability *h*^2^. Then we numerically solve the parameters (*α *and *θ*) based on the above equations. We set *p*(*D*) = 0.1 and *h*^2 ^= 0.02. The details are given in the supplementary document (Additional file 1).

## Authors' contributions

CY and XW contributed equally to this work. They developed the method and drafted the manuscript together. NT, QY, HX and WY initialized this work. WY finalized the manuscript. All authors read and approved the final manuscript.

## Supplementary Material

Additional file 1 **In the supplementary document (Additional le 1), we present the details of simulation**. We also give a brief introduction to log-linear models which are used in the main article. Finally, we provide full lists of the results identified from the WTCCC data sets. Click here for file
