# Supplementary material for: A hidden two-locus disease association pattern in genome-wide association studies
Source: BMC Bioinformatics. 2011 May 14;12:156. doi: 10.1186/1471-2105-12-156 (PMC3116488; doi:10.1186/1471-2105-12-156)
Supplement: Additional file 1 — In the supplementary document (Additional le 1), we present the details of simulation. We also give a brief introduction to log-linear models which are used in the main article. Finally, we provide full lists of the results identified from the WTCCC data sets. [file 1471-2105-12-156-S1.PDF]

Supplementary document
# – A hidden two-locus disease association pattern in genome-wide association studies

Can Yang*, Xiang Wan*, Qiang Yang,
Hong Xue, Nelson L.S. Tang and Weichuan Yu†

1 Simulation

We denote the major and minor alleles with capital letters (e.g., A or B) and lowercase letters (e.g., a or b), respectively. Let p_A and p_a be the frequencies of allele A and a at locus X_1, and let p_B and p_b be the frequencies of allele B and b at locus X_2. To simulate the linkage disequilibrium of loci X_1 and X_2, we need two conditional probabilities:

$$\begin{cases} p_0 & = p(X_2 = B | X_1 = A) \\ p_1 & = p(X_2 = B | X_1 = a) \end{cases} . \tag{1}$$

Now we have

$$\begin{cases} p_B & = p_A\, p_0 + p_a\, p_1 \\ p_b & = p_A\,(1 - p_0) + p_a\,(1 - p_1) \end{cases} . \tag{2}$$

We also have the frequencies of four haplotypes $\{AB, aB, Ab, ab\}$:

$$\begin{cases} p_{AB} & = p_A\, p_0 \\ p_{aB} & = p_a\, p_1 \\ p_{Ab} & = p_A\,(1 - p_0) \\ p_{ab} & = p_a\,(1 - p_1) \end{cases} . \tag{3}$$

The linkage disequilibrium measure $\mathcal{D}$ is defined as

$$\mathcal{D} = p_{AB}\, p_{ab} - p_{aB}\, p_{Ab}, \tag{4}$$

and the measure r^2 is defined as

$$r^2 = \frac{\mathcal{D}^2}{p_A\, p_a\, p_B\, p_b} = \frac{(p_{AB}\, p_{ab} - p_{aB}\, p_{Ab})^2}{p_A\, p_a\, p_B\, p_b}. \tag{5}$$

*These authors contribute equally to this work.
†corresponding author

	BB	Bb	bb
AA	$p_{AB}\,p_{AB}$	$2\,p_{AB}\,p_{Ab}$	$p_{Ab}\,p_{Ab}$
Aa	$2\,p_{AB}\,p_{aB}$	U	$2\,p_{Ab}\,p_{ab}$
aa	$p_{aB}\,p_{aB}$	$2\,p_{aB}\,p_{ab}$	$p_{ab}\,p_{ab}$

Table S1: The table for converting the haplotype frequency ito the genotype frequency. $U = 1 - \sum_{G_i \neq AaBb} p(G_i)$.

Plugging Eq. (3) into Eq. (5) yields

$$
\begin{aligned}
r^2 &= \frac{(p_A\,p_0\,p_a\,(1-p_1) - p_a\,p_1\,p_A\,(1-p_0))^2}{p_A\,p_a\,p_B\,p_b} \\
&= \frac{(p_A\,p_a\,(p_0 - p_1))^2}{p_A\,p_a\,p_B\,p_b} \\
&= \frac{p_A\,p_a\,(p_0 - p_1)^2}{p_B\,p_b}.
\end{aligned} \tag{6}
$$

Combining the above equation with Eq. (2) yields

$$
\begin{cases}
p_1 = p_B \pm |r| \sqrt{\frac{p_A\,p_B\,p_b}{p_a}} \\
p_0 = \frac{p_B - p_a\,p_1}{p_A}
\end{cases}. \tag{7}
$$

Notice that p_1 and p_0 should be valid probabilities. The sign of $\mathcal{D}$ (Eq. (4)) could be used to impose a positive correlation or negative correlation between these two loci in the population.

Given p_A, p_a, p_B, p_b and r^2, we can obtain p_1 and p_0 using Eq. (7). We can further obtain the frequencies of four haplotypes using Eq. (3) and obtain the frequencies of nine genotypes $p(G_i), i = 1, ..., 9$ (see Table 1). When $r^2 = 0$, Table 1 reduces to the genotype distribution generated by hardy-weinberg equilibrium.

Given the disease model, i.e., $p(D|G_i)$, the disease prevalence $p(D)$ and the genetic heritability h^2 are computed as

$$
\begin{cases}
p(D) = \sum_i p(D|G_i)p(G_i) \\
h^2 = \frac{\sum_i (p(D|G_i) - p(D))^2 p(G_i)}{p(D)(1-p(D))}
\end{cases}. \tag{8}
$$

In our simulation, we first specify the disease prevalence $p(D)$ and genetic heritability h^2. Then we numerically solve the model parameters (α and θ) based on Eq. (8). We set $p(D) = 0.1$ and $h^2 = 0.02$. The other unassociated loci are generated independently with the minor allele frequency uniformly distributed in [0.05,0.5].

2 Contingency tables and log-linear models

Given two loci X_p and X_q, a contingency table of X_p, X_q, Y will be used for testing the unfaithfulness associations between (X_p, X_q) and Y. The size of the contingency table is $I \times J \times K$, where $I = 3, J = 3, K = 2$. We use n_{ijk} to denote the observed count in the cell (i, j, k)

in the contingency table (Table 2), which is a realization of a random variable N_{ijk} assumed as Poisson-distributed in log-linear models. Clearly, we have $n = \sum_{i,j,k} n_{ijk}$. We use π_{ijk} to denote the probability that an observation falls in the cell (i, j, k). A natural constraint of π_{ijk} is $\sum_{i,j,k} \pi_{ijk} = 1$.

$Y = 0$	$X_q = 0$	$X_q = 1$	$X_q = 2$	$Y = 1$	$X_q = 0$	$X_q = 1$	$X_q = 2$
$X_p = 0$	n_{000}	n_{010}	n_{020}	$X_p = 0$	n_{001}	n_{011}	n_{021}
$X_p = 1$	n_{100}	n_{110}	n_{120}	$X_p = 1$	n_{101}	n_{111}	n_{121}
$X_p = 2$	n_{200}	n_{210}	n_{220}	$X_p = 2$	n_{201}	n_{211}	n_{221}

Table S2: The genotype counts in controls $Y = 0$) and cases ($Y = 1$) .

We use the dot convention to indicate the summation over a subscript, e.g., $n_{i..} = \sum_{j,k} n_{ijk}$ is the number of observations with $X_p = i$. Similarly, we have $n_{.j.} = \sum_{i,k} n_{ijk}$ and $n_{..k} = \sum_{i,j} n_{ijk}$. We also have $n_{ij.} = \sum_{k} n_{ijk}$, $n_{.jk} = \sum_{i} n_{ijk}$ and $n_{i.k} = \sum_{j} n_{ijk}$.

Log-linear models treat N_{ijk} as independent Poisson random variables with their means

$$\mu_{ijk} = n\pi_{ijk}. \tag{9}$$

The likelihood function is

$$f(\boldsymbol{\mu}) = \prod_{i,j,k} \frac{e^{-\mu_{ijk}} \mu_{ijk}^{n_{ijk}}}{n_{ijk}!}. \tag{10}$$

Correspondingly, the log-likelihood function is

$$L(\boldsymbol{\mu}) = \sum_{i,j,k} [n_{ijk} \log(\mu_{ijk}) - \mu_{ijk} - \log(n_{ijk}!)]. \tag{11}$$

To give a clear summary of log-linear models, we describe the following basic model structures:

1. Complete independence model M_C.

2. Block independence model M_B.

3. Partial independence model M_P.

4. Homogeneous association model M_H.

In the following, we begin with the complete independence model as an introduction to log-linear models, and then explain the block independence model and the partial independence model. Finally, we describe the homogeneous association model.

2.1 Complete independence model M_C

The simplest log-linear model structure is the complete independence model, in which the joint distribution of three variables is the product of three marginal distributions. It indicates that the joint distribution can be factorized completely. The corresponding hypothesis is

$$H_0^C : \pi_{ijk} = \pi_{i..}\pi_{.j.}\pi_{..k}. \tag{12}$$

Combining Eq. (9) and Eq. (12) yields

$$\log \mu_{ijk} = \log(n) + \log(\pi_{i..}) + \log(\pi_{.j.}) + \log(\pi_{..k}). \tag{13}$$

This gives the log-linear model with the additive structure:

$$\log \mu_{ijk} = \lambda + \lambda_i^{X_p} + \lambda_j^{X_q} + \lambda_k^{Y}, \tag{14}$$

where λ is a parameter which does not depend on either X_p or X_q, the parameter $\lambda_i^{X_p}$ denotes the effect of category i of X_p. Similar explanations are given for $\lambda_j^{X_q}$ and λ_k^{Y}, respectively. Notice that the superscript X_p is merely the label and does not represent the exponent. Pluging Eq. (14) into Eq. (10) and maximizing the likelihood yields the maximum likelihood estimator (MLE) of μ_{ijk}

$$\hat{\mu}_{ijk}^C = \frac{n_{i..}n_{.j.}n_{..k}}{n^2}. \tag{15}$$

2.2 Block independence model M_B

When the joint distribution can be factorized into blocks, the hypothesis is

$$H_0^B : \pi_{ijk} = \pi_{ij.}\pi_{..k}. \tag{16}$$

The corresponding log-linear model is

$$\log \mu_{ijk} = \lambda + \lambda_i^{X_p} + \lambda_j^{X_q} + \lambda_k^{Y} + \lambda_{ij}^{X_pX_q}. \tag{17}$$

Under this structure, the MLE of μ_{ijk} is

$$\hat{\mu}_{ijk}^B = \frac{n_{ij.}n_{..k}}{n}. \tag{18}$$

2.3 Partial independence model M_P

The joint distribution may be factorized when some variables are given. For example, given Y, the hypothesis is

$$H_0^P : \pi_{ijk} = \frac{\pi_{i.k}\pi_{.jk}}{\pi_{..k}}. \tag{19}$$

The corresponding log-linear model is

$$\log \mu_{ijk} = \lambda + \lambda_i^{X_p} + \lambda_j^{X_q} + \lambda_k^{Y} + \lambda_{ik}^{X_pY} + \lambda_{jk}^{X_qY}. \tag{20}$$

Then the MLE of μ_{ijk} is

$$\hat{\mu}_{ijk}^P = \frac{n_{i.k}n_{.jk}}{n_{..k}}. \tag{21}$$

2.4 Homogeneous association model M_H

An extension of Eq. (20) is to include all pairs:

$$\log \mu_{ijk} = \lambda + \lambda_i^{X_p} + \lambda_j^{X_q} + \lambda_k^{Y} + \lambda_{ij}^{X_pX_q} + \lambda_{ik}^{X_pY} + \lambda_{jk}^{X_qY}. \tag{22}$$

Exponentiating both sides, the cell probabilities π_{ijk} have the form

$$H_0^H : \pi_{ijk} = \psi_{ij}\phi_{ik}\omega_{jk}. \tag{23}$$

where ψ_{ij}, ϕ_{ik} and ω_{jk} are lower-order distributions. The name "homogeneous association" comes from the fact that the association between any two of three variables remains the same no matter what level the third [1].

Unfortunately, no closed-form expression exists for the three components in terms of marginal distributions of $\{\pi_{ijk}\}$. Iterative approaches, such as the Newton-Raphson method and iterative proportional fitting (IPF) [1], are needed to estimate the parameters.

2.5 Connection between log-linear models and logistic models

For convenience, we describe the equivalence between a log-linear model and its corresponding logistic model in this section. More details are provided in [1]. Here we use the homogeneous association model M_H (22) as an example. Its logit is

$$\begin{aligned}
&\log \frac{P(Y=1|X_p=i, X_q=j)}{P(Y=0|X_p=i, X_q=j)} \\
&= \log \frac{\mu_{ij1}}{\mu_{ij0}} \\
&= \log(\mu_{ij1}) - \log(\mu_{ij0}) \\
&= (\lambda + \lambda_i^{X_p} + \lambda_j^{X_q} + \lambda_1^{Y} + \lambda_{ij}^{X_pX_q} + \lambda_{i1}^{X_pY} + \lambda_{j1}^{X_qY}) \\
&\quad - (\lambda + \lambda_i^{X_p} + \lambda_j^{X_q} + \lambda_0^{Y} + \lambda_{ij}^{X_pX_q} + \lambda_{i0}^{X_pY} + \lambda_{j0}^{X_qY}) \\
&= (\lambda_1^{Y} - \lambda_0^{Y}) + (\lambda_{i1}^{X_pY} - \lambda_{i0}^{X_pY}) + (\lambda_{j1}^{X_qY} - \lambda_{j0}^{X_qY}).
\end{aligned} \tag{24}$$

The first term is a constant which does not depend on i or j. The second term only depends on the category i of X_p. The third term only depends on the category j of X_q. Therefore, this logit has the following form

$$\begin{aligned}
&\log \frac{P(Y=1|X_p=i, X_q=j)}{P(Y=0|X_p=i, X_q=j)} \\
&= (\lambda_1^{Y} - \lambda_0^{Y}) + (\lambda_{i1}^{X_pY} - \lambda_{i0}^{X_pY}) + (\lambda_{j1}^{X_qY} - \lambda_{j0}^{X_qY}) \\
&= \beta_0 + \beta_i^{X_p} + \beta_j^{X_q}.
\end{aligned} \tag{25}$$

Clearly, this is equivalent to the logistic model $\mathcal{M}_{1\bigoplus 2}$.

Based on similar inference, the equivalence between log-linear models and logistic models given in Table 2 in the main text can be obtained.

2.6 Likelihood ratio tests using log-linear models

Using Eq. (11), we can measure the association via the likelihood ratio test

$$\hat{L}_{M_H} - \hat{L}_{M_B} = \sum_{i,j,k} \left[n_{ijk} \log \frac{\hat{\mu}^H_{ijk}}{\hat{\mu}^B_{ijk}} - \hat{\mu}^H_{ijk} + \hat{\mu}^B_{ijk} \right]. \tag{26}$$

As Eq. (9) implies that

$$\sum_{i,j,k} \hat{\mu}^H_{ijk} = \sum_{i,j,k} \hat{\mu}^B_{ijk} = n, \tag{27}$$

Eq. (26) can be further reduced as

$$\hat{L}_{M_H} - \hat{L}_{M_B} = \sum_{i,j,k} \left[n_{ijk} \log \frac{\hat{\mu}^H_{ijk}}{\hat{\mu}^B_{ijk}} \right]. \tag{28}$$

Following the similar inference as mentioned above, we have

$$\hat{L}_{M_P} - \hat{L}_{M_B} = \sum_{i,j,k} \left[n_{ijk} \log \frac{\hat{\mu}^P_{ijk}}{\hat{\mu}^B_{ijk}} \right]. \tag{29}$$

3 Results

3.1 Q-Q plots of the WTCCC data sets

Here we provide the Q-Q plots of the association identified from the seven WTCCC data sets. There are more than 10^{10} χ^2 values for each data set, we can only record those χ^2 values greater than or equal to a threshold τ. We generate Q-Q plots as follows:

1. Let T_{ij} denote the χ^2 value of the joint regression analysis of SNPs i and j.
2. We obtain a set of χ^2 values by recording all $T_{ij} \geq \tau$, where the threshold $\tau = 30$. We denote this set by T_τ.
3. We generate a Q-Q plot using T_τ.

Figure 1 gives the Q-Q plots of the WTCCC data sets. We can see χ^2 values follow $\chi^2_{df=4}$ except the tails.

3.2 The full list of associations identified from WTCCC data sets

In the main text, we have connected some identified associations to publicly available results from other association studies. In this section, we provide the full list of associations identified from WTCCC data sets in Table 3-9.

The interaction P-values provided in the last column of Table 3-9 are based on the statistical definition of gene-gene interactions [2, 3]. Let $\hat{L}_{\mathcal{M}_{1\bigoplus 2}}$ and $\hat{L}_{\mathcal{M}_{full}}$ be the likelihood of $\mathcal{M}_{1\bigoplus 2}$

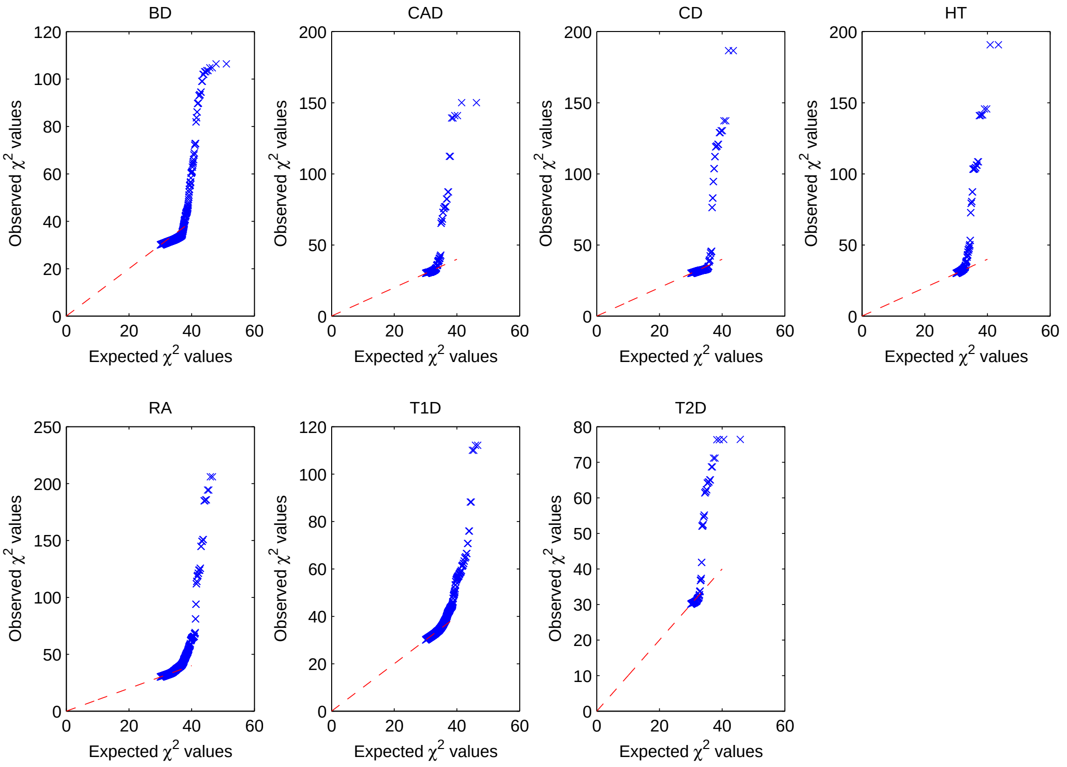

Figure 1: Q-Q plots of the WTCCC data sets

and $\mathcal{M}_{full}$ evaluated at their MLEs, respectively. $2(\hat{L}_{\mathcal{M}_{full}} - \hat{L}_{\mathcal{M}_{1\bigoplus 2}})$ follows χ^2 distribution with $df = 4$.

$$\mathcal{M}_{1\bigoplus 2} \; : \; \log \frac{P(Y=1|X_p=i, X_q=j)}{P(Y=0|X_p=i, X_q=j)} = \beta_0 + \beta_i^{X_p} + \beta_j^{X_q}. \tag{30}$$

$$\mathcal{M}_{full} \; : \; \log \frac{P(Y=1|X_p=i, X_q=j)}{P(Y=0|X_p=i, X_q=j)} = \beta_0 + \beta_i^{X_p} + \beta_j^{X_q} + \beta_{ij}^{X_p,X_q}. \tag{31}$$

Based on the statistical definition of gene-gene interactions, interaction effects can be either significant or nonsignificant when we discuss the associations masked by unfaithfullness. We have pointed this out in the discussion section of the main text.

SNP X_p				SNP X_p				Statistic	
SNP	Chr	Position	Single-locus P-value	SNP	Chr	Position	Single-locus P-value	Unfaithfulness association P-value	Interaction P-value
rs2349400	2	5358596	0.075	rs1453783	2	5368662	0.049	$< 1.0 \times 10^{-16}$	0.861
rs10929488	2	5367555	0.039	rs1453783	2	5368662	0.049	$< 1.0 \times 10^{-16}$	0.208
rs1453783	2	5368662	0.049	rs4349322	2	5372971	0.125	7.294×10^{-14}	4.662×10^{-4}
rs1453783	2	5368662	0.049	rs6728135	2	5374777	0.141	$< 1.0 \times 10^{-16}$	0.326
rs1453783	2	5368662	0.049	rs10929491	2	5376586	0.084	$< 1.0 \times 10^{-16}$	0.043
rs246571	5	71840906	0.616	rs417769	5	71847234	0.004	1.359×10^{-12}	0.741
rs10970619	9	31967010	0.215	rs10970672	9	31999054	8.522×10^{-4}	5.224×10^{-13}	0.007
rs17278280	9	31976303	0.713	rs10970672	9	31999054	8.522×10^{-4}	1.697×10^{-12}	0.095
rs11849674	14	59757776	0.575	rs7154773	14	59818871	0.001	$< 1.0 \times 10^{-16}$	$< 1.0 \times 10^{-16}$
rs10148587	14	59758913	0.634	rs7154773	14	59818871	0.001	9.992×10^{-16}	4.741×10^{-14}
rs10137732	14	59758962	0.435	rs7154773	14	59818871	0.001	$< 1.0 \times 10^{-16}$	$< 1.0 \times 10^{-16}$
rs6573298	14	59775222	0.364	rs7154773	14	59818871	0.001	$< 1.0 \times 10^{-16}$	3.277×10^{-4}
rs7145505	14	59775328	0.401	rs7154773	14	59818871	0.001	$< 1.0 \times 10^{-16}$	0.001
rs8019531	14	59777164	0.528	rs7154773	14	59818871	0.001	$< 1.0 \times 10^{-16}$	7.473×10^{-10}
rs11628587	14	59777184	0.227	rs7154773	14	59818871	0.001	$< 1.0 \times 10^{-16}$	0.008
rs11628628	14	59777299	0.231	rs7154773	14	59818871	0.001	$< 1.0 \times 10^{-16}$	1.609×10^{-4}
rs8011227	14	59788029	0.704	rs7154773	14	59818871	0.001	$< 1.0 \times 10^{-16}$	2.729×10^{-7}
rs7158657	14	59794810	0.943	rs7154773	14	59818871	0.001	$< 1.0 \times 10^{-16}$	0.641
rs10142834	14	59804716	0.958	rs7154773	14	59818871	0.001	$< 1.0 \times 10^{-16}$	0.490
rs1887103	14	59812972	0.928	rs7154773	14	59818871	0.001	$< 1.0 \times 10^{-16}$	0.243
rs1657117	16	1843652	0.909	rs1742431	16	1845875	0.098	1.365×10^{-12}	0.212

Table S3: The identified associations involving two SNPs from the CD data set.

SNP X_p				SNP X_p				Statistic	
SNP	Chr	Position	Single-locus P-value	SNP	Chr	Position	Single-locus P-value	Unfaithfulness association P-value	Interaction P-value
rs4654792	1	22345978	0.056	rs909812	1	22348672	0.003	1.529×10^{-12}	0.021
rs6683655	1	185168259	0.166	rs12137523	1	185173706	0.004	1.055×10^{-14}	0.937
rs12137523	1	185173706	0.004	rs4396083	1	185179880	0.197	1.509×10^{-12}	0.101
rs2349400	2	5358596	0.207	rs1453783	2	5368662	0.222	$< 1.0 \times 10^{-16}$	0.989
rs10929488	2	5367555	0.256	rs1453783	2	5368662	0.222	$< 1.0 \times 10^{-16}$	0.999
rs1453783	2	5368662	0.222	rs6728135	2	5374777	0.219	7.550×10^{-15}	0.416
rs1453783	2	5368662	0.222	rs10929491	2	5376586	0.233	2.331×10^{-15}	0.654
rs6846200	4	43048682	0.139	rs4277811	4	43049698	0.671	5.296×10^{-14}	0.376
rs6846200	4	43048682	0.139	rs4309873	4	43051416	0.680	6.564×10^{-13}	0.962
rs945238	6	84222575	0.016	rs6903322	6	84225687	0.067	1.743×10^{-14}	0.061
rs6415311	7	146023635	0.003	rs10251563	7	146057413	0.504	2.354×10^{-12}	0.234
rs4732651	8	28637742	0.746	rs2237812	8	28641955	0.333	1.373×10^{-13}	8.134×10^{-4}
rs11248546	10	125232273	0.024	rs913525	10	125243227	0.574	1.041×10^{-12}	0.485
rs6573298	14	59775222	0.270	rs7154773	14	59818871	0.063	7.772×10^{-16}	0.169
rs7145505	14	59775328	0.273	rs7154773	14	59818871	0.063	1.443×10^{-15}	0.216
rs8019531	14	59777164	0.169	rs7154773	14	59818871	0.063	8.882×10^{-16}	6.883×10^{-15}
rs11628587	14	59777184	0.150	rs7154773	14	59818871	0.063	1.110×10^{-16}	0.150
rs11628628	14	59777299	0.126	rs7154773	14	59818871	0.063	$< 1.0 \times 10^{-16}$	0.317
rs8011227	14	59788029	0.395	rs7154773	14	59818871	0.063	$< 1.0 \times 10^{-16}$	0.003
rs7158657	14	59794810	0.196	rs7154773	14	59818871	0.063	$< 1.0 \times 10^{-16}$	0.555
rs10142834	14	59804716	0.191	rs7154773	14	59818871	0.063	$< 1.0 \times 10^{-16}$	0.179
rs1887103	14	59812972	0.193	rs7154773	14	59818871	0.063	$< 1.0 \times 10^{-16}$	0.458
rs7162070	15	37708210	0.867	rs16969478	15	37718245	0.160	5.551×10^{-15}	0.175
rs1876853	15	37708459	0.903	rs16969478	15	37718245	0.160	2.310×10^{-13}	0.072
rs8029602	15	37717832	0.853	rs16969478	15	37718245	0.160	5.274×10^{-14}	0.741
rs16969475	15	37718161	0.823	rs16969478	15	37718245	0.160	1.259×10^{-13}	0.839

Table S4: The identified associations involving two SNPs from the CA data set.

SNP X_p				SNP X_p				Statistic	
SNP	Chr	Position	Single-locus P-value	SNP	Chr	Position	Single-locus P-value	Unfaithfulness association P-value	Interaction P-value
rs668860	1	85148483	0.053	rs10873672	1	85162747	0.245	4.885×10^{-15}	0.550
rs668860	1	85148483	0.053	rs6691970	1	85162941	0.216	6.217×10^{-15}	0.483
rs7417737	1	226774798	0.841	rs3811480	1	226775937	0.024	3.331×10^{-16}	0.012
rs7414802	1	226775010	0.833	rs3811480	1	226775937	0.024	3.775×10^{-15}	0.019
rs2349400	2	5358596	0.202	rs1453783	2	5368662	0.135	$< 1.0 \times 10^{-16}$	0.524
rs10929488	2	5367555	0.211	rs1453783	2	5368662	0.135	$< 1.0 \times 10^{-16}$	0.388
rs1453783	2	5368662	0.135	rs6728135	2	5374777	0.083	$< 1.0 \times 10^{-16}$	0.090
rs1453783	2	5368662	0.135	rs10929491	2	5376586	0.085	$< 1.0 \times 10^{-16}$	0.141
rs13028177	2	36148101	0.477	rs6543990	2	36151725	0.628	1.645×10^{-12}	0.039
rs7570865	2	44216708	0.002	rs6544738	2	44216891	0.501	1.557×10^{-13}	0.347
rs17046061	2	54775681	0.724	rs17046067	2	54779147	0.003	7.192×10^{-13}	0.215
rs1110998	2	217169458	0.588	rs2738290	2	217177759	0.012	2.100×10^{-12}	0.012
rs2738287	2	217175342	0.647	rs2738290	2	217177759	0.012	2.406×10^{-13}	0.002
rs4688938	4	5634102	0.002	rs4586871	4	5634450	0.682	6.661×10^{-16}	0.966
rs2935260	5	54480637	0.934	rs2992406	5	54480740	0.059	$< 1.0 \times 10^{-16}$	0.994
rs13179425	5	81870050	0.129	rs7727362	5	81870550	0.496	7.772×10^{-16}	0.880
rs11167615	5	152548555	0.365	rs2080950	5	152552163	0.005	4.136×10^{-13}	0.564
rs12515563	5	152935825	0.391	rs1552835	5	152939962	0.064	4.009×10^{-13}	0.989
rs1552837	5	152939862	0.339	rs1552835	5	152939962	0.064	3.830×10^{-14}	0.995
rs1552835	5	152939962	0.064	rs17519558	5	152940512	0.869	1.110×10^{-16}	0.990
rs1552835	5	152939962	0.064	rs17591636	5	152940808	0.468	2.082×10^{-12}	0.857
rs2438083	6	1222371	0.997	rs977674	6	1222702	0.016	$< 1.0 \times 10^{-16}$	0.220
rs2438083	6	1222371	0.997	rs977673	6	1222715	0.014	$< 1.0 \times 10^{-16}$	0.563
rs1729549	6	131547858	0.043	rs1190806	6	131567377	0.765	8.530×10^{-13}	0.032
rs985882	7	19348745	0.685	rs985881	7	19348795	0.060	3.268×10^{-13}	0.370
rs2677051	7	93009176	0.599	rs2677048	7	93009694	0.004	1.110×10^{-16}	0.556
rs10253608	7	157968536	0.195	rs10266006	7	157973801	0.349	4.030×10^{-14}	0.967
rs12113120	7	157969556	0.291	rs10266006	7	157973801	0.349	2.205×10^{-12}	0.537
rs10266006	7	157973801	0.349	rs3793181	7	157981303	0.143	8.105×10^{-15}	0.924
rs10266006	7	157973801	0.349	rs6459895	7	157982346	0.211	4.756×10^{-13}	0.692
rs10266006	7	157973801	0.349	rs12698265	7	157988773	0.190	6.151×10^{-14}	0.906
rs2447183	8	120422159	0.678	rs2469997	8	120422448	0.257	$< 1.0 \times 10^{-16}$	0.317
rs2469996	8	120422192	0.800	rs2469997	8	120422448	0.257	$< 1.0 \times 10^{-16}$	0.061
rs2469997	8	120422448	0.257	rs6469823	8	120423165	0.820	$< 1.0 \times 10^{-16}$	1
rs2469997	8	120422448	0.257	rs2447179	8	120424956	0.783	$< 1.0 \times 10^{-16}$	0.969
rs2469997	8	120422448	0.257	rs2447178	8	120425369	0.703	$< 1.0 \times 10^{-16}$	0.971
rs2469997	8	120422448	0.257	rs2470002	8	120426605	0.815	$< 1.0 \times 10^{-16}$	0.971
rs2469997	8	120422448	0.257	rs2470025	8	120433908	0.833	$< 1.0 \times 10^{-16}$	8.959×10^{-4}
rs2469997	8	120422448	0.257	rs2447169	8	120434244	0.771	$< 1.0 \times 10^{-16}$	0.970
rs2469997	8	120422448	0.257	rs2470026	8	120434293	0.861	5.773×10^{-15}	1.130×10^{-4}
rs2469997	8	120422448	0.257	rs2447168	8	120434794	0.785	$< 1.0 \times 10^{-16}$	3.581×10^{-4}
rs2469997	8	120422448	0.257	rs2470040	8	120454633	0.756	5.995×10^{-15}	1.552×10^{-4}
rs4934884	10	37964698	0.053	rs4934888	10	37979171	0.012	1.332×10^{-15}	0.432
rs4934888	10	37979171	0.012	rs1208771	10	38112505	0.121	4.075×10^{-14}	0.368
rs12805895	11	37664444	0.107	rs1381428	11	37665229	0.539	2.159×10^{-12}	0.040
rs11237746	11	78556141	0.011	rs11237747	11	78561641	0.920	1.221×10^{-13}	0.853
rs11177914	12	68546631	0.783	rs11177919	12	68551936	0.069	8.027×10^{-14}	0.015
rs10872999	14	45741665	0.898	rs10483596	14	45810659	0.023	1.033×10^{-12}	0.630
rs886889	17	12744926	0.485	rs10521202	17	12755289	0.005	5.551×10^{-16}	0.769

Table S5: The identified associations involving two SNPs fromthe BD data set.

SNP X_p				SNP X_p				Statistic	
SNP	Chr	Position	Single-locus P-value	SNP	Chr	Position	Single-locus P-value	Unfaithfulness association P-value	Interaction P-value
rs4654792	1	22345978	0.081	rs909812	1	22348672	0.086	1.093×10^{-12}	0.028
rs13420028	2	133021838	0.614	rs10188442	2	133022971	0.444	6.678×10^{-13}	0.791
rs959880	3	184571315	0.797	rs2314349	3	184573800	0.003	1.166×10^{-14}	0.991
rs2314349	3	184573800	0.003	rs906719	3	184573846	0.585	2.032×10^{-14}	0.964
rs2314349	3	184573800	0.003	rs2314348	3	184574032	0.791	5.116×10^{-13}	0.010
rs2314349	3	184573800	0.003	rs2089588	3	184574176	0.782	1.266×10^{-14}	0.331
rs11941043	4	34171656	0.305	rs7667516	4	34172794	0.644	1.221×10^{-12}	0.369
rs7668845	4	34172736	0.239	rs7667516	4	34172794	0.644	5.507×10^{-14}	0.984
rs17113078	5	151677459	0.156	rs1363415	5	151696888	0.233	1.157×10^{-12}	0.627
rs2978169	8	95092674	0.009	rs3018857	8	95093002	0.456	1.574×10^{-12}	0.424
rs2447183	8	120422159	0.578	rs2469997	8	120422448	0.724	3.664×10^{-14}	0.048
rs2469996	8	120422192	0.599	rs2469997	8	120422448	0.724	4.996×10^{-15}	0.074
rs2469997	8	120422448	0.724	rs6469823	8	120423165	0.544	8.882×10^{-16}	1
rs2469997	8	120422448	0.724	rs2447179	8	120424956	0.559	$< 1.0 \times 10^{-16}$	0.651
rs2469997	8	120422448	0.724	rs2447178	8	120425369	0.570	$< 1.0 \times 10^{-16}$	0.971
rs2469997	8	120422448	0.724	rs2470002	8	120426605	0.622	2.109×10^{-15}	0.072
rs2469997	8	120422448	0.724	rs2470025	8	120433908	0.557	3.109×10^{-15}	0.111
rs2469997	8	120422448	0.724	rs2447169	8	120434244	0.574	1.110×10^{-16}	0.972
rs2469997	8	120422448	0.724	rs2470026	8	120434293	0.551	2.220×10^{-14}	0.369
rs2469997	8	120422448	0.724	rs2447168	8	120434794	0.628	6.120×10^{-13}	6.800×10^{-6}
rs2469997	8	120422448	0.724	rs2470040	8	120454633	0.487	9.337×10^{-14}	0.036
rs10817300	9	97085490	0.005	rs10981362	9	97094963	0.399	2.381×10^{-12}	0.992
rs10817300	9	97085490	0.005	rs7863530	9	97096318	0.417	1.508×10^{-12}	0.577
rs10817300	9	97085490	0.005	rs6478048	9	97102307	0.389	3.796×10^{-13}	0.859
rs10816425	9	106557265	0.564	rs2035783	9	106558834	0.052	7.413×10^{-13}	0.082
rs9942941	9	106558689	0.550	rs2035783	9	106558834	0.052	1.485×10^{-13}	0.389
rs11849674	14	59757776	0.056	rs7154773	14	59818871	0.031	$< 1.0 \times 10^{-16}$	1.019×10^{-8}
rs10148587	14	59758913	0.114	rs7154773	14	59818871	0.031	$< 1.0 \times 10^{-16}$	4.756×10^{-7}
rs10137732	14	59758962	0.051	rs7154773	14	59818871	0.031	$< 1.0 \times 10^{-16}$	1.282×10^{-10}
rs6573298	14	59775222	0.206	rs7154773	14	59818871	0.031	$< 1.0 \times 10^{-16}$	0.176
rs7145505	14	59775328	0.188	rs7154773	14	59818871	0.031	$< 1.0 \times 10^{-16}$	0.456
rs8019531	14	59777164	0.368	rs7154773	14	59818871	0.031	1.110×10^{-16}	2.742×10^{-14}
rs11628587	14	59777184	0.168	rs7154773	14	59818871	0.031	$< 1.0 \times 10^{-16}$	0.291
rs11628628	14	59777299	0.153	rs7154773	14	59818871	0.031	$< 1.0 \times 10^{-16}$	0.348
rs8011227	14	59788029	0.216	rs7154773	14	59818871	0.031	$< 1.0 \times 10^{-16}$	0.002
rs7158657	14	59794810	0.362	rs7154773	14	59818871	0.031	$< 1.0 \times 10^{-16}$	0.762
rs10142834	14	59804716	0.346	rs7154773	14	59818871	0.031	$< 1.0 \times 10^{-16}$	0.256
rs1887103	14	59812972	0.249	rs7154773	14	59818871	0.031	$< 1.0 \times 10^{-16}$	0.959
rs2757527	14	99730686	0.010	rs2757528	14	99730830	0.459	2.220×10^{-16}	0.525
rs2757527	14	99730686	0.010	rs2766696	14	99731073	0.453	6.106×10^{-15}	0.022
rs8085875	18	43913365	0.343	rs6417101	18	43926817	0.001	$< 1.0 \times 10^{-16}$	5.548×10^{-4}
rs2300390	21	34891385	0.460	rs12482676	21	34892582	0.061	2.442×10^{-15}	0.718

Table S6: The identified associations involving two SNPs from the HT data set.

SNP X_p				SNP X_p				Statistic	
SNP	Chr	Position	Single-locus P-value	SNP	Chr	Position	Single-locus P-value	Unfaithfulness association P-value	Interaction P-value
rs1343295	1	163038411	0.004	rs7543540	1	163039943	0.867	$< 1.0 \times 10^{-16}$	2.631×10^{-7}
rs1343295	1	163038411	0.004	rs6693244	1	163040481	0.883	$< 1.0 \times 10^{-16}$	1.700×10^{-7}
rs6425407	1	173782656	0.400	rs12732164	1	173787788	0.128	1.266×10^{-12}	0.038
rs6425407	1	173782656	0.400	rs11583509	1	173788674	0.113	9.565×10^{-13}	0.035
rs2808250	1	197324204	0.068	rs2809345	1	197327920	0.538	9.437×10^{-15}	0.184
rs2349400	2	5358596	0.215	rs1453783	2	5368662	0.185	$< 1.0 \times 10^{-16}$	0.128
rs10929488	2	5367555	0.177	rs1453783	2	5368662	0.185	$< 1.0 \times 10^{-16}$	0.245
rs1453783	2	5368662	0.185	rs6728135	2	5374777	0.277	2.998×10^{-15}	0.612
rs1453783	2	5368662	0.185	rs10929491	2	5376586	0.228	4.441×10^{-16}	0.202
rs7570544	2	108306655	0.004	rs7599646	2	108310861	0.735	1.906×10^{-12}	0.762
rs7601781	2	153639741	0.804	rs4664635	2	153656443	0.110	4.585×10^{-13}	1.976×10^{-14}
rs2136152	3	7257402	0.003	rs1605705	3	7265044	0.013	$< 1.0 \times 10^{-16}$	1.172×10^{-10}
rs4686119	3	7257739	0.004	rs1605705	3	7265044	0.013	$< 1.0 \times 10^{-16}$	$< 1.0 \times 10^{-16}$
rs908465	3	7258715	0.005	rs1605705	3	7265044	0.013	$< 1.0 \times 10^{-16}$	$< 1.0 \times 10^{-16}$
rs1605705	3	7265044	0.013	rs9809928	3	7277350	0.005	$< 1.0 \times 10^{-16}$	$< 1.0 \times 10^{-16}$
rs1605705	3	7265044	0.013	rs1396402	3	7305517	0.003	$< 1.0 \times 10^{-16}$	$< 1.0 \times 10^{-16}$
rs2290408	4	1057097	0.729	rs1010342	4	1058649	0.140	2.210×10^{-12}	4.584×10^{-9}
rs6846200	4	43048682	0.063	rs4277811	4	43049698	0.589	1.110×10^{-16}	0.662
rs6834503	4	115128779	0.882	rs10005635	4	115130782	0.101	$< 1.0 \times 10^{-16}$	2.721×10^{-4}
rs9275765	6	32797302	0.014	rs7453920	6	32837990	8.606×10^{-4}	8.339×10^{-13}	0.073
rs9275772	6	32797481	0.015	rs7453920	6	32837990	8.606×10^{-4}	8.541×10^{-13}	0.023
rs9275793	6	32798005	0.013	rs7453920	6	32837990	8.606×10^{-4}	5.587×10^{-13}	0.081
rs10947844	6	40061050	0.069	rs10947857	6	40219493	0.003	$< 1.0 \times 10^{-16}$	3.011×10^{-6}
rs9296318	6	40061909	0.436	rs10947857	6	40219493	0.003	$< 1.0 \times 10^{-16}$	9.953×10^{-4}
rs4594945	6	40062137	0.156	rs10947857	6	40219493	0.003	$< 1.0 \times 10^{-16}$	7.006×10^{-14}
rs10456478	6	40068291	0.442	rs10947857	6	40219493	0.003	$< 1.0 \times 10^{-16}$	0.102
rs2894387	6	40070178	0.370	rs10947857	6	40219493	0.003	$< 1.0 \times 10^{-16}$	2.443×10^{-4}
rs10807222	6	40082317	0.535	rs10947857	6	40219493	0.003	$< 1.0 \times 10^{-16}$	0.003
rs1007026	6	40147009	0.471	rs10947857	6	40219493	0.003	$< 1.0 \times 10^{-16}$	1.348×10^{-8}
rs10947854	6	40174494	0.697	rs10947857	6	40219493	0.003	$< 1.0 \times 10^{-16}$	4.848×10^{-13}
rs10807224	6	40180690	0.599	rs10947857	6	40219493	0.003	$< 1.0 \times 10^{-16}$	0.022
rs6912289	6	40187497	0.577	rs10947857	6	40219493	0.003	$< 1.0 \times 10^{-16}$	3.274×10^{-8}
rs10947855	6	40204867	0.826	rs10947857	6	40219493	0.003	$< 1.0 \times 10^{-16}$	$< 1.0 \times 10^{-16}$
rs2091113	6	40207363	0.558	rs10947857	6	40219493	0.003	$< 1.0 \times 10^{-16}$	2.040×10^{-6}
rs10947856	6	40219405	0.625	rs10947857	6	40219493	0.003	$< 1.0 \times 10^{-16}$	4.268×10^{-7}
rs10947857	6	40219493	0.003	rs940357	6	40220640	0.520	$< 1.0 \times 10^{-16}$	2.012×10^{-5}
rs9293855	6	72678221	0.828	rs1891698	6	72689749	0.004	$< 1.0 \times 10^{-16}$	2.886×10^{-8}
rs945238	6	84222575	0.675	rs6903322	6	84225687	0.324	7.577×10^{-13}	0.002
rs945238	6	84222575	0.675	rs6454329	6	84228251	0.408	4.484×10^{-13}	0.032
rs945238	6	84222575	0.675	rs10943933	6	84228649	0.308	1.221×10^{-12}	0.281
rs9942681	7	125781690	0.819	rs2106311	7	125823341	0.021	$< 1.0 \times 10^{-16}$	0.001
rs12154335	7	125786322	0.862	rs2106311	7	125823341	0.021	$< 1.0 \times 10^{-16}$	0.002
rs1361990	7	125812180	0.904	rs2106311	7	125823341	0.021	$< 1.0 \times 10^{-16}$	3.627×10^{-7}
rs2106311	7	125823341	0.021	rs2299476	7	125838281	0.973	$< 1.0 \times 10^{-16}$	3.924×10^{-7}
rs2106311	7	125823341	0.021	rs6467092	7	125856946	0.969	$< 1.0 \times 10^{-16}$	3.941×10^{-5}
rs2106311	7	125823341	0.021	rs728600	7	125872759	0.966	$< 1.0 \times 10^{-16}$	0.002
rs2106311	7	125823341	0.021	rs1419484	7	125880552	0.928	$< 1.0 \times 10^{-16}$	8.276×10^{-6}
rs2106311	7	125823341	0.021	rs7792592	7	125889892	0.980	$< 1.0 \times 10^{-16}$	2.492×10^{-5}
rs4732651	8	28637742	0.105	rs2237812	8	28641955	0.997	2.222×10^{-12}	1.736×10^{-5}
rs10883365	10	101277754	0.278	rs1548964	10	101279643	0.027	4.707×10^{-13}	3.504×10^{-9}
rs10883367	10	101277980	0.403	rs1548964	10	101279643	0.027	4.611×10^{-13}	5.256×10^{-10}
rs1548964	10	101279643	0.027	rs1548962	10	101279725	0.308	1.110×10^{-15}	2.808×10^{-7}
rs7896275	10	114071985	0.050	rs4256909	10	114083575	0.021	$< 1.0 \times 10^{-16}$	1.110×10^{-16}
rs6421986	11	211659	0.964	rs6598060	11	233987	0.147	2.348×10^{-12}	6.646×10^{-5}
rs6598060	11	233987	0.147	rs7116130	11	234129	0.784	4.876×10^{-13}	2.404×10^{-4}

Continued on next page

Table 7 – continued from previous page									
SNP X_p				SNP X_p				Statistic	
SNP	Chr	Position	Single-locus P-value	SNP	Chr	Position	Single-locus P-value	Unfaithfulness association P-value	Interaction P-value
rs6598060	11	233987	0.147	rs1128322	11	234197	0.980	$< 1.0 \times 10^{-16}$	0.120
rs4376999	12	24512236	0.175	rs10842357	12	24519700	0.001	2.465×10^{-14}	0.138
rs4963762	12	24513427	0.171	rs10842357	12	24519700	0.001	$< 1.0 \times 10^{-16}$	0.057
rs4765066	12	124763828	0.083	rs4765292	12	124765818	0.466	1.873×10^{-12}	0.075
rs279937	13	102502078	0.589	rs279936	13	102502609	0.176	$< 1.0 \times 10^{-16}$	0.976
rs12427557	13	103167268	0.008	rs7328544	13	103167449	0.167	$< 1.0 \times 10^{-16}$	4.307×10^{-7}
rs10137732	14	59758962	0.247	rs7154773	14	59818871	0.087	4.253×10^{-13}	1.110×10^{-16}
rs6573298	14	59775222	0.142	rs7154773	14	59818871	0.087	$< 1.0 \times 10^{-16}$	0.005
rs7145505	14	59775328	0.173	rs7154773	14	59818871	0.087	$< 1.0 \times 10^{-16}$	0.003
rs8019531	14	59777164	0.059	rs7154773	14	59818871	0.087	$< 1.0 \times 10^{-16}$	2.036×10^{-12}
rs11628587	14	59777184	0.112	rs7154773	14	59818871	0.087	$< 1.0 \times 10^{-16}$	0.010
rs11628628	14	59777299	0.091	rs7154773	14	59818871	0.087	$< 1.0 \times 10^{-16}$	2.143×10^{-5}
rs8011227	14	59788029	0.213	rs7154773	14	59818871	0.087	$< 1.0 \times 10^{-16}$	6.277×10^{-5}
rs7158657	14	59794810	0.143	rs7154773	14	59818871	0.087	$< 1.0 \times 10^{-16}$	0.272
rs10142834	14	59804716	0.103	rs7154773	14	59818871	0.087	$< 1.0 \times 10^{-16}$	0.280
rs1887103	14	59812972	0.117	rs7154773	14	59818871	0.087	$< 1.0 \times 10^{-16}$	0.949
rs12452792	17	67861219	0.802	rs11077601	17	67861735	0.268	$< 1.0 \times 10^{-16}$	0.065
rs11077601	17	67861735	0.268	rs9916746	17	67867163	0.628	8.947×10^{-13}	0.039
rs13370227	18	73724640	0.011	rs7407082	18	73729102	0.350	3.331×10^{-16}	0.353

Table S7: The identified associations involving two SNPs fromthe RA data set.

SNP X_p				SNP X_p				Statistic	
SNP	Chr	Position	Single-locus P-value	SNP	Chr	Position	Single-locus P-value	Unfaithfulness association P-value	Interaction P-value
rs2349400	2	5358596	0.134	rs1453783	2	5368662	0.315	$< 1.0 \times 10^{-16}$	0.313
rs10929488	2	5367555	0.144	rs1453783	2	5368662	0.315	$< 1.0 \times 10^{-16}$	0.442
rs1453783	2	5368662	0.315	rs6728135	2	5374777	0.126	7.372×10^{-14}	0.633
rs1453783	2	5368662	0.315	rs10929491	2	5376586	0.153	6.817×10^{-14}	0.061
rs7637028	3	72757615	0.158	rs7639455	3	72757722	0.299	$< 1.0 \times 10^{-16}$	0.213
rs2438074	6	1214690	0.993	rs2438083	6	1222371	0.010	1.688×10^{-12}	0.044
rs2496292	6	1219617	0.929	rs2438083	6	1222371	0.010	1.577×10^{-14}	7.868×10^{-4}
rs2438083	6	1222371	0.010	rs977674	6	1222702	0.785	$< 1.0 \times 10^{-16}$	0.010
rs2438083	6	1222371	0.010	rs977673	6	1222715	0.776	$< 1.0 \times 10^{-16}$	0.021
rs1058318	6	30620142	0.074	rs1264432	6	30670000	0.802	1.221×10^{-15}	0.029
rs1058318	6	30620142	0.074	rs2252745	6	30687294	0.840	1.326×10^{-12}	8.215×10^{-4}
rs6908994	6	31306688	0.002	rs2394963	6	31359441	0.004	2.442×10^{-13}	0.294
rs6908994	6	31306688	0.002	rs16899205	6	31374340	0.005	5.851×10^{-13}	0.228
rs6908994	6	31306688	0.002	rs16899207	6	31374366	0.006	9.523×10^{-13}	0.298
rs2736172	6	31698877	0.006	rs805297	6	31730585	0.047	1.256×10^{-13}	0.551
rs6573298	14	59775222	0.471	rs7154773	14	59818871	0.029	3.331×10^{-16}	0.099
rs7145505	14	59775328	0.416	rs7154773	14	59818871	0.029	$< 1.0 \times 10^{-16}$	0.181
rs8019531	14	59777164	0.137	rs7154773	14	59818871	0.029	$< 1.0 \times 10^{-16}$	3.948×10^{-13}
rs11628587	14	59777184	0.384	rs7154773	14	59818871	0.029	$< 1.0 \times 10^{-16}$	0.330
rs11628628	14	59777299	0.363	rs7154773	14	59818871	0.029	$< 1.0 \times 10^{-16}$	0.257
rs8011227	14	59788029	0.622	rs7154773	14	59818871	0.029	$< 1.0 \times 10^{-16}$	2.776×10^{-6}
rs7158657	14	59794810	0.256	rs7154773	14	59818871	0.029	$< 1.0 \times 10^{-16}$	0.872
rs10142834	14	59804716	0.283	rs7154773	14	59818871	0.029	$< 1.0 \times 10^{-16}$	0.174
rs1887103	14	59812972	0.223	rs7154773	14	59818871	0.029	$< 1.0 \times 10^{-16}$	0.653
rs6574988	14	87629745	0.002	rs4581640	14	87630108	0.774	$< 1.0 \times 10^{-16}$	0.244
rs6574988	14	87629745	0.002	rs2401743	14	87630502	0.695	$< 1.0 \times 10^{-16}$	0.898
rs2757527	14	99730686	0.017	rs2757528	14	99730830	0.937	1.895×10^{-12}	0.819

Table S8: The identified unfaithfulness associations involving two SNPs from the T1D data set.

SNP X_p				SNP X_p				Statistic	
SNP	Chr	Position	Single-locus P-value	SNP	Chr	Position	Single-locus P-value	Unfaithfulness association P-value	Interaction P-value
rs3790857	1	63823887	0.008	rs2269238	1	63829558	0.974	1.904×10^{-13}	0.295
rs1365752	1	80568982	0.491	rs1896250	1	80569215	0.001	1.802×10^{-12}	0.466
rs691531	1	86957299	9.420×10^{-4}	rs1208054	1	86983009	0.250	$< 1.0 \times 10^{-16}$	1.227×10^{-7}
rs6713149	2	2432966	0.596	rs4853937	2	2435048	0.002	3.508×10^{-14}	0.849
rs2349400	2	5358596	0.205	rs1453783	2	5368662	0.246	$< 1.0 \times 10^{-16}$	0.995
rs10929488	2	5367555	0.242	rs1453783	2	5368662	0.246	$< 1.0 \times 10^{-16}$	0.990
rs1453783	2	5368662	0.246	rs4349322	2	5372971	0.231	3.886×10^{-15}	0.004
rs1453783	2	5368662	0.246	rs6728135	2	5374777	0.208	$< 1.0 \times 10^{-16}$	0.671
rs1453783	2	5368662	0.246	rs10929491	2	5376586	0.149	$< 1.0 \times 10^{-16}$	0.740
rs266403	3	6456002	0.999	rs266404	3	6456514	0.038	9.067×10^{-13}	0.096
rs6826705	4	1112464	0.705	rs2127908	4	1118755	0.206	$< 1.0 \times 10^{-16}$	0.082
rs6826705	4	1112464	0.705	rs4974627	4	1119805	0.148	$< 1.0 \times 10^{-16}$	0.952
rs7378252	4	21411058	0.005	rs9291430	4	21421717	0.339	1.110×10^{-15}	0.053
rs7378252	4	21411058	0.005	rs2018175	4	21430677	0.467	3.531×10^{-14}	0.016
rs7378252	4	21411058	0.005	rs10031539	4	21435498	0.302	1.110×10^{-16}	0.002
rs7378252	4	21411058	0.005	rs17498679	4	21438015	0.267	$< 1.0 \times 10^{-16}$	0.063
rs6531531	4	32719183	0.002	rs10022638	4	32725553	0.266	1.366×10^{-14}	0.446
rs6531531	4	32719183	0.002	rs6848027	4	32741310	0.361	3.553×10^{-15}	0.246
rs1173196	5	4869811	0.062	rs1173198	5	4871616	0.477	9.279×10^{-13}	0.011
rs1173196	5	4869811	0.062	rs816471	5	4881722	0.482	1.572×10^{-12}	0.027
rs1173196	5	4869811	0.062	rs816478	5	4886867	0.421	1.459×10^{-12}	0.027
rs7791862	7	47516496	0.014	rs7792409	7	47516602	0.113	1.299×10^{-14}	1.295×10^{-11}
rs7791862	7	47516496	0.014	rs7792551	7	47516674	0.295	9.992×10^{-16}	2.637×10^{-9}
rs7791862	7	47516496	0.014	rs7792432	7	47516725	0.364	9.992×10^{-16}	2.234×10^{-9}
rs1513921	7	108200381	0.180	rs4730350	7	108201181	0.876	4.297×10^{-14}	0.336
rs10253608	7	157968536	0.016	rs10266006	7	157973801	0.199	9.754×10^{-13}	0.098
rs12113120	7	157969556	0.007	rs10266006	7	157973801	0.199	3.333×10^{-13}	0.345
rs10949739	7	157971558	0.040	rs10266006	7	157973801	0.199	1.470×10^{-12}	0.007
rs10266006	7	157973801	0.199	rs3793181	7	157981303	0.011	3.921×10^{-13}	0.171
rs10266006	7	157973801	0.199	rs6459895	7	157982346	0.010	2.510×10^{-13}	0.453
rs10266006	7	157973801	0.199	rs12698265	7	157988773	0.011	1.250×10^{-12}	0.038
rs6481484	10	28163077	0.555	rs7078534	10	28163722	0.470	1.594×10^{-12}	0.691
rs10490898	10	28898895	0.373	rs6481534	10	28907403	0.065	1.366×10^{-12}	0.966
rs201892	11	32639267	0.050	rs1486581	11	32680527	0.873	7.772×10^{-16}	0.562
rs201892	11	32639267	0.050	rs10767965	11	32752668	0.857	4.151×10^{-13}	3.727×10^{-4}
rs9971746	12	110800082	0.094	rs16941759	12	110830641	0.005	9.490×10^{-13}	0.930
rs7162070	15	37708210	0.406	rs16969478	15	37718245	0.129	2.288×10^{-12}	0.764
rs8029602	15	37717832	0.575	rs16969478	15	37718245	0.129	1.599×10^{-12}	0.412
rs8094798	18	67929854	0.009	rs9960006	18	67942342	0.273	1.307×10^{-13}	0.617
rs8094798	18	67929854	0.009	rs997133	18	67943715	0.314	7.117×10^{-14}	0.631
rs8094798	18	67929854	0.009	rs10514039	18	67955521	0.225	2.245×10^{-13}	0.353
rs1555322	20	33312595	0.060	rs2425037	20	33319843	0.498	5.261×10^{-13}	0.771

Table S9: The identified associations involving two SNPs fromthe T2D data set.

References

[1] A. Agresti. *Categorical Data Analysis.* Wiley Series in Probability and Statistics. Wiley and Sons INC., second edition, 2002.

[2] H.J. Cordell. Epistasis: what it means, what it doesn't mean, and statistical methods to detect it in humans. *Human Molecular Genetics*, 11:2463–2468, 2002.

[3] H.J. Cordell. Detecting gene-gene interactions that underlie human diseases. *Nature Reviews Genetics*, 10:392–404, 2009.
